# Simultaneous semen and urine biomonitoring of neonicotinoids and associations with semen quality in men

**DOI:** 10.3389/fendo.2026.1871388

**Published:** 2026-09-28

**Authors:** Li-Jun Lei, Lu Zhang, Qing-Shuang Yang, Zi-Jun Zhou, Jiao-Jing Liu, Fang-Di Dong, Zhi-Wei Xue, Ling-Yuan Wu, Zhi-Peng Cheng, Ping-Ping Wang, Yu-Bin Ding, Hui-Jun Yang

**Affiliations:** 1 Assisted Reproductive Center, Key Laboratory of Birth Regulation and Control Technology of National Health Commission of China, Shandong Provincial Key Medical and Health Discipline of Reproductive Medicine, Shandong Provincial Maternal and Child Health Care Hospital Affiliated to Qingdao University, Jinan, China; 2 Department of Reproductive Toxicology, Joint International Research Laboratory of Reproduction and Development of the Ministry of Education of China, School of Public Health, Chongqing Medical University, Chongqing, China; 3 Ministry of Education Key Laboratory of Pollution Processes and Environmental Criteria, College of Environmental Science and Engineering, Nankai University, Tianjin, China; 4 National Engineering Research Center of Pesticide, College of Chemistry, Nankai University, Tianjin, China; 5 Department of Pharmacology, Hunan Provincial Key Laboratory of the Research and Development of Novel Pharmaceutical Preparations School of Pharmacology, Changsha Medical University, Changsha, China

**Keywords:** biomonitoring, neonicotinoid insecticide, semen, semen quality, urine

## Abstract

**Background:**

Neonicotinoid insecticides (NEOs) are among the most widely used insecticides worldwide, yet their presence in seminal plasma and potential implications for male reproductive health remain poorly characterized. Urine—the conventional biomonitoring matrix—may not fully reflect concentrations in reproductive-relevant tissues, highlighting the need for direct seminal biomonitoring.

**Methods:**

In a cross-sectional study of 179 men attending a reproductive technology clinic in Jinan, China, 18 NEO parent compounds and metabolites (m-NEOs) were simultaneously quantified in urine (n = 179) and semen (n = 173) samples using UPLC-MS/MS. Among the 18 target analytes, 14 were quantified in at least one biological matrix. Associations between NEO exposure and semen quality parameters were assessed using multivariable linear regression, restricted cubic spline models, Bayesian kernel machine regression (BKMR), and quantile-based g-computation (QGC).

**Results:**

Among the successfully quantified analytes, 11 NEOs and m-NEOs were detected in urine (>75% detection frequency) versus four in semen; urinary and seminal concentrations were weakly correlated (r = –0.02 to 0.38), suggesting matrix-specific exposure profiles. Urinary 5-hydroxy-imidacloprid (5-OH-IMI) was associated with sperm concentration, total count and normal morphology. Seminal desnitro-imidacloprid (DN-IMI) and imidacloprid-urea (IMI-urea) were negatively associated with sperm concentration, total sperm count, and motility. Mixture analyses revealed a 27% reduction in sperm concentration per one-quartile increase in seminal NEO exposure (QGC: –27%, 95% CI: –43% to –7%), with DN-IMI identified as the major contributor to the observed mixture association (BKMR posterior inclusion probability (PIP) = 0.913). Age-stratified analyses suggested age-related differences in associations between NEO exposure and semen quality.

**Conclusions:**

Seminal NEO concentrations, which were not adequately reflected by urinary biomarkers, were associated with lower sperm concentration, highlighting semen as a potentially informative matrix for reproductive risk assessment. These findings underscore that assessments relying solely on urinary biomarkers may not fully capture reproductive-relevant NEO exposure profiles. Longitudinal studies incorporating hormone profiling and functional sperm assessments are needed to further clarify causal pathways and inform regulatory thresholds.

## 1 Introduction

Infertility is a growing global health concern, affecting approximately 8–17% of couples worldwide, with male factors contributing to nearly half of all cases (1). Epidemiological data spanning five decades document a significant secular decline in sperm concentration and total sperm count in men from North America, Europe, and Australia, raising urgent questions about the environmental drivers of this trend (2, 3). Among putative environmental contributors, neonicotinoid insecticides (NEOs) have emerged as a class of particular concern. Since their commercial introduction in the 1990s, NEOs have become among the most widely used insecticides worldwide, displacing organophosphates and pyrethroids across agricultural, veterinary, and residential applications and currently accounting for approximately one-quarter of the global insecticide market share (4, 5). Their systemic distribution and long environmental half-life mean that humans are continuously exposed through dietary intake, inhalation, dermal absorption, and dust ingestion (6, 7). NEOs and neonicotinoid metabolites (m-NEOs) have now been detected at measurable concentrations in human urine (8), serum (9), hair (10), and follicular fluid (11), demonstrating widespread human exposure. Despite this ubiquity, the consequences of NEO exposure for male reproductive health in humans remain incompletely understood.

A substantial body of experimental evidence supports NEO reproductive toxicity. In rodent models, imidacloprid (IMI) and acetamiprid (ACE) exposure has been shown to disrupt testicular morphology, impair sperm motility and count, reduce serum testosterone, and induce structural damage to seminiferous tubules and Leydig cells (12–14). These adverse effects may be mediated, at least in part, by m-NEOs: desnitro-imidacloprid (DN-IMI), a major reductive metabolite of IMI, exhibits markedly higher mammalian receptor affinity than the parent compound and has been independently implicated in testicular toxicity (15, 16). In human epidemiological studies, higher urinary NEO concentrations have been associated with decreased serum testosterone and altered semen parameters (17), providing preliminary evidence for endocrine-disrupting effects in men. However, existing human studies are limited by small sample sizes, cross-sectional designs, and near-exclusive reliance on urinary biomarkers that may not reflect the exposure levels at reproductive-relevant compartments. This fundamental translational gap — between robust animal evidence and sparse, matrix-limited human data — points to the need for a more rigorous biomonitoring approach.

A key methodological limitation of existing human studies is their reliance on urine as the sole biomonitoring matrix. While urinary m-NEO concentrations reflect recent systemic exposure and renal excretion, they do not necessarily represent the biologically relevant local concentrations to which germ cells and somatic cells within the testis are actually exposed (18). The blood–testis barrier (BTB) is one of the most selective biological barriers in the mammalian body, tightly regulating the entry of xenobiotics from systemic circulation into the seminiferous epithelium and seminal plasma (19). BTB permeability to environmental chemicals is governed by compound-specific physicochemical properties, including lipophilicity, molecular weight, and plasma protein binding affinity, meaning that seminal concentrations of individual NEOs and m-NEOs cannot be reliably predicted from urinary measurements alone. Unlike urine, which primarily reflects systemic exposure during absorption, metabolism, and elimination processes, seminal plasma represents the extracellular microenvironment surrounding spermatozoa and may provide additional information regarding reproductive tract-specific exposure. Recent evidence from our group demonstrated distinct distribution patterns of NEOs and their metabolites between paired urine and semen samples, with several compounds showing preferential enrichment in semen, highlighting the potential value of semen as a reproductive-relevant exposure matrix (20). Previous studies have demonstrated that concentrations of environmental chemicals in seminal plasma may not fully correspond to urinary biomarkers, indicating that these matrices capture distinct aspects of internal exposure profiles (21). Therefore, seminal plasma may serve as a complementary biomonitoring matrix that more closely reflects the biologically relevant exposure scenario for male reproductive tissues, particularly for endocrine-disrupting chemicals with potential effects on sperm function and reproductive outcomes. Seminal plasma therefore represents a distinct and arguably more biologically proximate exposure matrix for assessing reproductive risk — analogous to how follicular fluid has been used as a target-tissue matrix for evaluating female reproductive toxicity (11). Yet to date, only one published study has quantified NEOs directly in human seminal plasma, reporting a negative association between imidacloprid-olefin and sperm motility parameters in a limited number of participants (22). That study examined only a single parent compound and its olefin metabolite, included no concurrent urinary measurements, and lacked the statistical power to evaluate mixture effects. No study has simultaneously collected paired urine and semen from the same men to directly compare NEO concentrations across matrices, validate the biomonitoring utility of each approach, or determine whether seminal NEO concentrations convey reproductive risk information beyond what urinary biomarkers alone can provide.

A further limitation of the existing literature is the near-exclusive use of single-pollutant regression models that evaluate each NEO compound in isolation. Human exposure commonly involves multiple NEO parent compounds and their metabolites originating from shared environmental and dietary sources, as suggested by environmental monitoring and biomonitoring studies (6, 23, 24). These structurally related compounds may exhibit partially overlapping toxicological pathways, although their combined effects on human reproductive health remain insufficiently characterized. Single-pollutant models are susceptible to confounding from omitted co-exposures and cannot capture synergistic or antagonistic interactions among mixture components, nor the nonlinear dose–response relationships that may operate across a biologically realistic range of exposures (25, 26). Methodological advances in environmental epidemiology have produced purpose-built tools — including Bayesian kernel machine regression (BKMR) and quantile-based g-computation (QGC) — that enable simultaneous, flexible estimation of overall mixture effects, nonlinear exposure–response functions, pollutant–pollutant interactions, and the relative contribution of individual components to the total mixture association (25, 26). Applying these approaches to a comprehensive panel of co-occurring NEOs is essential for moving beyond single-compound toxicology toward a realistic, integrated assessment of mixture-level reproductive risk in men.

This study was designed to address these converging gaps. Using simultaneously collected urine and semen samples from 179 men attending an assisted reproductive technology clinic in Jinan, China, we pursued three specific objectives: (1) to simultaneously quantify 18 parent NEOs and m-NEOs in both urine and semen, enabling the first direct cross-matrix comparison of detection frequencies, concentration distributions, and urine–semen concordance within the same individuals; (2) to evaluate the independent associations of urinary and seminal NEO concentrations with a comprehensive panel of semen quality parameters, assessing whether target-tissue seminal concentrations provide exposure-outcome information distinct from urinary biomarkers; and (3) to quantify the joint mixture effects of co-occurring NEOs on semen quality using BKMR and QGC, identify the relative contributions of individual NEO components to the overall mixture association, and explore whether age modifies NEO-associated reproductive susceptibility. The study design, analytical methods, and statistical framework are described in the following section.

## 2 Materials and methods

### 2.1 Chemicals and reagents

Analytical standards of parent NEOs, metabolites of NEOs (m-NEOs) and isotopically labeled internal standards (ISs) were obtained from Sigma-Aldrich (St. Louis, MO, USA). Five isotope-labeled internal standards—acetamiprid-*d_3_* (ACE-*d_3_*), clothianidin-*d_3_* (CLO-*d_3_*), imidacloprid-*d_3_* (IMI-*d_3_*), thiacloprid-(thiazolidin-ring-*d_4_*) (THCP-*d_4_*), and thiamethoxam-*d_4_* (THM-*d_4_*)—were used for quantification. All standards were of > 97% purity. Other reagents, including formic acid, acetic acid, β-glucuronidase, methanol, ethyl acetate, and Milli-Q water, were obtained from commercial suppliers. Detailed information on the target analytes and internal standards is provided in **Supplementary Table S1**.

### 2.2 Study population and sample collection

The study population consisted of male partners of infertile couples seeking assisted reproductive technology (ART) services at an ART clinic in Shandong Province. Couples sought medical care for infertility attributed to male, female, combined, or unexplained factors. Therefore, the participating men represented a broad spectrum of reproductive health status, rather than exclusively male-factor infertility cases. Some men had common male infertility conditions—such as oligozoospermia, asthenozoospermia, teratozoospermia, or varicocele—but these diagnoses were not used as exclusion criteria unless they fulfilled one of the specific exclusion conditions listed below. Urine and semen samples were collected between March and November 2024. Inclusion criteria were: (1) age > 20 years; and (2) provision of at least one biological sample (urine or semen). Exclusion criteria were: (1) confirmed azoospermia; (2) pituitary adenoma or hyperprolactinemia; (3) use of medications known to affect semen quality (e.g., anabolic steroids, testosterone preparations, chemotherapeutic agents) within the past 3 months; and (4) chromosomal abnormalities or other known hereditary diseases affecting fertility. All participants were instructed to maintain 2–7 days of sexual abstinence before semen collection. Semen samples were collected on the day of oocyte retrieval from their partners, ensuring a highly consistent sampling time point across participants. This standardized protocol minimized variability related to abstinence duration and collection timing and thus reduced the potential influence of sampling time on the detection results.

Urine samples were collected in sterile polypropylene containers during the same clinical visit and stored at –20 °C immediately after collection. Semen samples were obtained via masturbation after 2–7 days of sexual abstinence and collected in sterile polypropylene containers. Following complete liquefaction at 37 °C within 60 minutes, semen was aliquoted and frozen at –80 °C until further analysis. A total of 179 urine samples and 173 semen samples were collected, among which 146 participants provided paired semen and urine samples. All participants were given full informed consent. The study protocol was authorized by the Ethics Committee of Shandong Provincial Maternal and Child Health Care Hospital (No. 2024-116).

### 2.3 Semen analysis

All analyses were conducted by trained technicians in accordance with World Health Organization (27) guidelines. Following liquefaction at 37 °C for up to 60 minutes, semen volume was determined by weight, assuming a density of 1.0 g/mL. Sperm concentration was measured by loading 10 μL of thoroughly mixed semen into a clean Makler chamber and examined under an optical microscope. Total sperm count was calculated by multiplying semen volume by sperm concentration. Progressive and non-progressive motility were assessed using an ocular grid, and total motility was defined as their sum. Sperm morphology was evaluated under a phase contrast microscope from sperm samples fixed in buffered 2% formaldehyde solution and recorded as the proportion of spermatozoa with normal morphology. All assessments were performed using standardized equipment, with internal quality control procedures in place to ensure inter-technician consistency.

### 2.4 Sample extraction and instrumental analysis

Urine and semen samples were extracted based on a previously reported method (28). Briefly, 1.0 mL of each sample was enzymatically hydrolyzed with β-glucuronidase and spiked with isotope-labeled internal standards. Target compounds were extracted using ethyl acetate, concentrated under a gentle nitrogen stream, and reconstituted in methanol prior to instrumental analysis.

NEOs and m-NEOs were quantified using a Waters ACQUITY UPLC-TQS-MS/MS system equipped with electrospray ionization in positive mode. Quantification was performed using multiple reaction monitoring (MRM). Detailed chromatographic conditions, mass spectrometric parameters, and transitions are provided in **Supplementary Methods S1** and **Supplementary Table S2**.

### 2.5 Quality assurance and quality control

Method validation followed standard quality assurance and quality control (QA/QC) procedures, including procedural and instrumental blanks, ten-point calibration curves, determination of method detection and quantification limits (29, 30), spiked recovery and precision experiments, and assessment of matrix effects (31); detailed procedures are provided in **Supplementary Methods S2**. Quantification was performed using ten-point calibration curves (0–200 ng/mL), which showed excellent linearity for all analytes, with correlation coefficients (R²) ranging from 0.991 to 1.000 in both matrices (**Supplementary Methods S2**; **Supplementary Figure S1**; **Supplementary Table S2**). The limits of detection (LODs) for the analytes ranged from 0.003 to 0.089 ng/mL in urine and 0.021 to 1.087 ng/mL in semen (**Supplementary Table S2**). Among the 18 initially targeted NEOs and m-NEOs, four compounds (Descyano-acetamiprid, Flonicamid, Thiamethoxam, and Thiacloprid-amide) were excluded from further quantification due to analytical limitations, including high background signals or poor calibration performance. Therefore, 14 analytes were successfully quantified in at least one biological matrix. For analytes with concentrations below the LOD, values were treated as non-detectable (ND) and imputed as follows: (i) LOD/2 for compounds with detection frequencies (DFs) > 50%, or (ii) *f* × LOD for those with DFs < 50%, where *f* represents the proportion of detectable samples (32, 33). Urinary NEO concentrations were further corrected for dilution using specific gravity (SG) according to the formula: *C_SG_* = *C* × [(*SG*_mean_ – 1)/(*SG* – 1)], where *C_SG_* is the SG-adjusted concentration, *C* is the measured concentration, *SG* is the specific gravity of the individual sample, and *SGmean* is the arithmetic mean of *SG* across the study population (34). SG-corrected concentrations were used for all subsequent analyses.

### 2.6 Covariate assessment

Participants completed structured questionnaires covering demographic characteristics, lifestyle factors, abstinence duration, and occupational status. Potential confounders were identified based on previous literature and directed acyclic graph (DAG) analysis (**Supplementary Figure S2**; **Supplementary Text S1**).

The final regression models were adjusted for age, body mass index (BMI), education level, smoking status, season of sample collection, abstinence duration, and occupational exposure likelihood.

### 2.7 Statistical analysis

Descriptive analyses were performed to summarize participant characteristics and NEO concentrations. Continuous variables were presented as medians (P25, P75), and categorical variables as numbers (percentages). Differences in participant characteristics among urine-only, semen-only, and paired urine–semen groups were assessed using the Kruskal–Wallis test or Pearson’s chi-square test, as appropriate. NEOs with detection frequencies (DFs) > 70% were included in subsequent analyses. NEO concentrations and semen quality parameters were natural log-transformed before regression analyses due to non-normal distributions.

Multivariable linear regression models were used to evaluate associations between NEO concentrations (in urine and semen) and semen quality parameters. Exposure variables were analyzed as continuous variables and quartiles, with the lowest quartile as the reference group. Trend tests and interaction analyses were performed to assess dose–response relationships and potential effect modification by age. Restricted cubic spline (RCS) models were further applied to explore nonlinear exposure–response relationships.

Bayesian kernel machine regression (BKMR) was conducted to assess the joint, nonlinear, and interactive effects of NEO mixtures on semen quality parameters (26). Detailed model specifications are provided in **Supplementary Methods S3**. NEO concentrations were log-transformed and standardized before modeling, and all models were adjusted for relevant covariates. Model convergence was evaluated using standard diagnostic criteria (**Supplementary Table S3**; **Supplementary Figure S3**).

Quantile-based g-computation (QGC) was additionally applied to estimate the overall mixture effects of NEOs and the relative contributions of individual components (25). Regression coefficients and 95% confidence intervals were obtained using bootstrap resampling (1,000 iterations).

Furthermore, sensitivity analyses were conducted to assess the robustness of the findings. These analyses included: (1) restricting analyses to participants with paired urine–semen samples; (2) standardizing ln-transformed NEO concentrations using z-scores; (3) additionally adjusting for semen volume in models evaluating seminal NEOs; and (4) re-imputing values below the LOD using the LOD/√2 method to evaluate the impact of alternative imputation approaches.

All statistical analyses were performed using R software (version 4.4.1; R Foundation for Statistical Computing, Vienna, Austria) and Python (version 3.8.0; Python Software Foundation). A two-sided P value <0.05 was considered statistically significant.

## 3 Result

### 3.1 Study population characteristics

The demographic characteristics and semen quality parameters of the participants across the three sample groups—urine only (n = 179), semen only (n = 173), and paired urine–semen samples (n = 146)—are summarized in **Table 1**. No statistically significant differences were observed in any demographic or semen quality variables across the groups (all *P* ≥ 0.609). The median age was 34 years (interquartile Range [IQR]: 31.0–38.0), and BMI values were similar across groups (median: 26.0–26.1 kg/m²). Semen quality indicators were also similar across groups, including volume (median: 3.54–3.60 mL), concentration (54–55 ×10^6^/mL), total sperm count (175–180 ×10^6^), progressive motility (32.0–32.4%), total motility (52.1–54.0%), and normal morphology (4.0% in all groups). These findings indicate that participants in the three groups were comparable in terms of baseline characteristics and semen quality.

**Table 1:** Demographic characteristics of the participants.

Characteristic	Urine (N = 179)	Semen (N = 173)	Paired (N = 146)	*P* value^a^
**Age, years, Median (Q1, Q3)**	34.0 (31.0, 38.0)	34.0 (31.0, 38.0)	34.0 (31.0, 38.0)	0.993
**BMI, kg/m^2^, Median (Q1, Q3)**	26.0 (24.0, 28.4)	26.1 (24.0, 28.4)	26.1 (23.9, 28.4)	> 0.999
**Season, n (%)**				0.839
Autumn	14 (7.82%)	18 (10.40%)	14 (9.59%)	
Spring	85 (47.49%)	74 (42.77%)	69 (47.26%)	
Summer	80 (44.69%)	81 (46.82%)	63 (43.15%)	
**Occupation, n (%)**				0.871
High	73 (40.78%)	70 (40.46%)	63 (43.15%)	
Low	106 (59.22%)	103 (59.54%)	83 (56.85%)	
**Education, n (%)**				0.904
College above	118 (65.92%)	111 (64.16%)	93 (63.70%)	
High school below	61 (34.08%)	62 (35.84%)	53 (36.30%)	
**Smoke, n (%)**				0.825
No	138 (77.09%)	129 (74.57%)	109 (74.66%)	
Yes	41 (22.91%)	44 (25.43%)	37 (25.34%)	
**Abstinence, n (%)**				0.985
<3 days	25 (13.97%)	25 (14.45%)	23 (15.75%)	
>5 days	56 (31.28%)	52 (30.06%)	42 (28.77%)	
3–5 days	98 (54.75%)	96 (55.49%)	81 (55.48%)	
**Volume, mL, Median (Q1, Q3)**	3.54 (2.63, 4.55)	3.60 (2.79, 4.64)	3.57 (2.70, 4.56)	0.642
**Concentration, 10^6^/mL, Median (Q1, Q3)**	54 (31, 81)	55 (33, 84)	55 (33, 81)	0.832
**Sperm count, 10^6^, Median (Q1, Q3)**	175 (102, 294)	180 (113, 355)	176 (108, 335)	0.609
**Progressive motility, %, Median (Q1, Q3)**	32 (18, 46)	32 (21, 44)	32 (21, 46)	0.968
**Total motility, %, Median (Q1, Q3)**	52 (34, 64)	53 (35, 63)	54 (35, 64)	0.934
**Sperm morphology, %, Median (Q1, Q3)**	4.00 (3.00, 6.00)	4.00 (3.00, 6.00)	4.00 (3.00, 6.00)	0.772

a One-way analysis of variance (ANOVA) for continuous variables and Pearson’s chi-square test for categorical variables. BMI, body mass index.

### 3.2 NEO concentrations in urine and semen

**Table 2** summarizes the detection frequencies and concentrations of NEOs and m-NEOs in urine and semen samples. Among the 18 compounds analyzed, 11 were detected in urine with detection frequencies exceeding 75%, namely desnitro-dinotefuran (DN, 99.44%), clothianidin (CLO, 99.44%), DN-IMI (98.32%), desnitro-imidacloprid-olefin (DN-IMI-olefin, 93.30%), IMI (92.74%), imidaclothiz (IMIT, 91.06%), imidacloprid-urea (IMI-urea, 86.59%), dinotefuran-urea (UF, 84.92%), 5-OH-IMI (82.68%), DM-ACE (95.53%), and ACE (100.00%). In contrast, only a limited number of compounds exhibited high detection frequencies in semen, notably DN (99.42%), DN-IMI (88.44%), IMI-urea (86.13%), and ACE (93.06%). DN presented the highest median concentration in both matrices, with a median of 3.25 ng/mL in semen and 1.04 ng/mL in urine. IMI-urea concentrations were substantially higher in semen (median: 0.96 ng/mL) than in urine (median: 0.10 ng/mL). Several other metabolites, including DN-IMI-olefin and 5-OH-IMI, were commonly detected in urine 95.5% and 82.7%, respectively), but were less frequently detected in semen (60.7% and 28.3%, respectively). A number of compounds—such as descyano-acetamiprid (Descynao-ACE), thiacloprid-amide (THCP-amide), flonicamid (FLO) and thiamethoxam (THM)—were not detected in either matrix. Correlation analyses revealed weak associations between NEO concentrations in urine and semen (range, –0.02 to 0.38) (**Supplementary Figure S4**).

**Table 2:** Descriptive statistics of concentrations of NEOs and metabolites of NEOs (m-NEOs) detected in urine and semen (ng/ml).

Chemicals (ng/ml)	Urine					Semen				
	DFs (%)	Median	*P*25	*P*75	Range	DFs (%)	Median	*P*25	*P*75	Range
DN	99.44	1.04	0.77	1.34	ND ~ 8.03	99.42	3.25	2.65	4.20	ND ~ 8.79
UF	84.92	0.26	0.14	0.52	ND ~ 12.23	43.35	ND	ND	0.41	ND ~ 1.09
Descynao-ACE	-	-	-	-	-	-	-	-	-	-
DN-IMI-olefin	93.30	0.11	0.07	0.19	ND ~ 1.82	46.24	ND	ND	0.09	ND ~ 0.26
DM-ACE	95.53	0.57	0.37	2.12	ND ~ 22.74	60.69	0.13	ND	0.25	ND ~ 3.45
DN-IMI	98.32	0.30	0.06	3.41	ND ~ 22.25	88.44	0.10	0.04	0.21	ND ~ 1.26
IMI-urea	86.59	0.10	0.06	0.14	ND ~ 0.84	86.13	0.96	0.58	1.39	ND ~ 15.16
THCP-amide	-	-	-	-	-	-	-	-	-	-
5-OH-IMI	82.68	0.21	0.11	0.34	ND ~ 28.43	28.32	ND	ND	0.94	ND ~ 5.36
DM-THM	40.78	ND	ND	0.47	ND ~ 4.83	-	-	-	-	-
DNT	-	-	-	-	-	46.82	ND	ND	1.92	ND ~ 9.07
ACE	100.00	0.09	0.06	0.12	0.04~0.80	93.06	0.54	0.19	1.07	ND ~ 17.88
FLO	-	-	-	-	-	-	-	-	-	-
CLO	99.44	0.47	0.25	0.84	ND ~ 46.75	63.58	0.24	ND	0.40	ND ~ 2.12
THCP	63.13	0.02	ND	0.04	ND ~ 0.72	-	-	-	-	-
IMI	92.74	0.13	0.09	0.28	ND ~ 3.59	19.08	ND	ND	ND	ND ~ 3.05
IMIT	91.06	0.09	0.06	0.13	ND ~ 7.02	43.35	ND	ND	0.29	ND ~ 1.06
THM	-	-	-	-	-	-	-	-	-	-

“–” indicates compounds that were excluded from quantification in this study due to analytical limitations (e.g., high background signals or poor calibration curve fitting) or were not detected in a given biological matrix. Among the 18 target analytes, 14 were successfully quantified in at least one biological matrix (urine or semen). Four compounds (FLO, THM, Descyno-ACE, THCP-amide) were excluded from quantification due to analytical limitations. DFs: detection frequencies, DN: desnitro-dinotefuran, UF: dinotefuran-urea, Descynao-ACE: descyano-acetamiprid, DM-ACE: desmethyl-acetamiprid, DN-IMI-olefin: desnitro-imidacloprid-olefin, DN-IMI: desnitro-imidacloprid, IMI-urea: imidacloprid-urea, THCP-amide: thiacloprid-amide, 5-OH-IMI: 5-hydroxy-imidacloprid, DM-THM: desmethyl-thiamethoxam, DNT: dinotefuran, ACE: acetamiprid, FLO: flonicamid, CLO: clothianidin, THCP: thiacloprid, IMI: imidacloprid, IMIT: imidaclothiz, THM: thiamethoxam.

### 3.3 Associations of NEO concentrations with semen quality

The associations of individual NEO concentrations in urine or semen with semen quality are summarized in **Figure 1**; **Supplementary Table S4**. After adjusting for potential confounders, we observed significant associations between urinary and seminal NEOs and semen quality parameters. In models treating urinary NEOs as continuous variables, a positive association was observed between DN-IMI-olefin and semen volume (β = 0.07, 95% CI, 0.01 to 0.14, *P* = 0.029), while the other ten compounds showed no statistically significant associations. Urinary 5-OH-IMI concentrations were negatively associated with sperm concentration (β = –0.13, 95% CI, –0.25 to –0.01, *P* = 0.036), total sperm count (β = –0.13, 95% CI, –0.26 to –0.01, *P* = 0.044), and the percentage of morphologically normal sperm (β = –0.07, 95% CI, –0.13 to –0.01, *P* = 0.024). DN concentrations were inversely associated with progressive motility (β = –0.19, 95% CI, –0.35 to –0.02, *P* = 0.029) and total motility (β = –0.13, 95% CI, –0.26 to –0.01, *P* = 0.044). In addition, urinary DN-IMI-olefin was inversely associated with total motility (β = –0.09, 95% CI, –0.17 to –0.01, *P* = 0.028). No other associations reached statistical significance. When NEO concentrations were categorized into quartiles, significant exposure–response trends were observed for several associations (**Supplementary Figure S5**).

**Figure 1:**
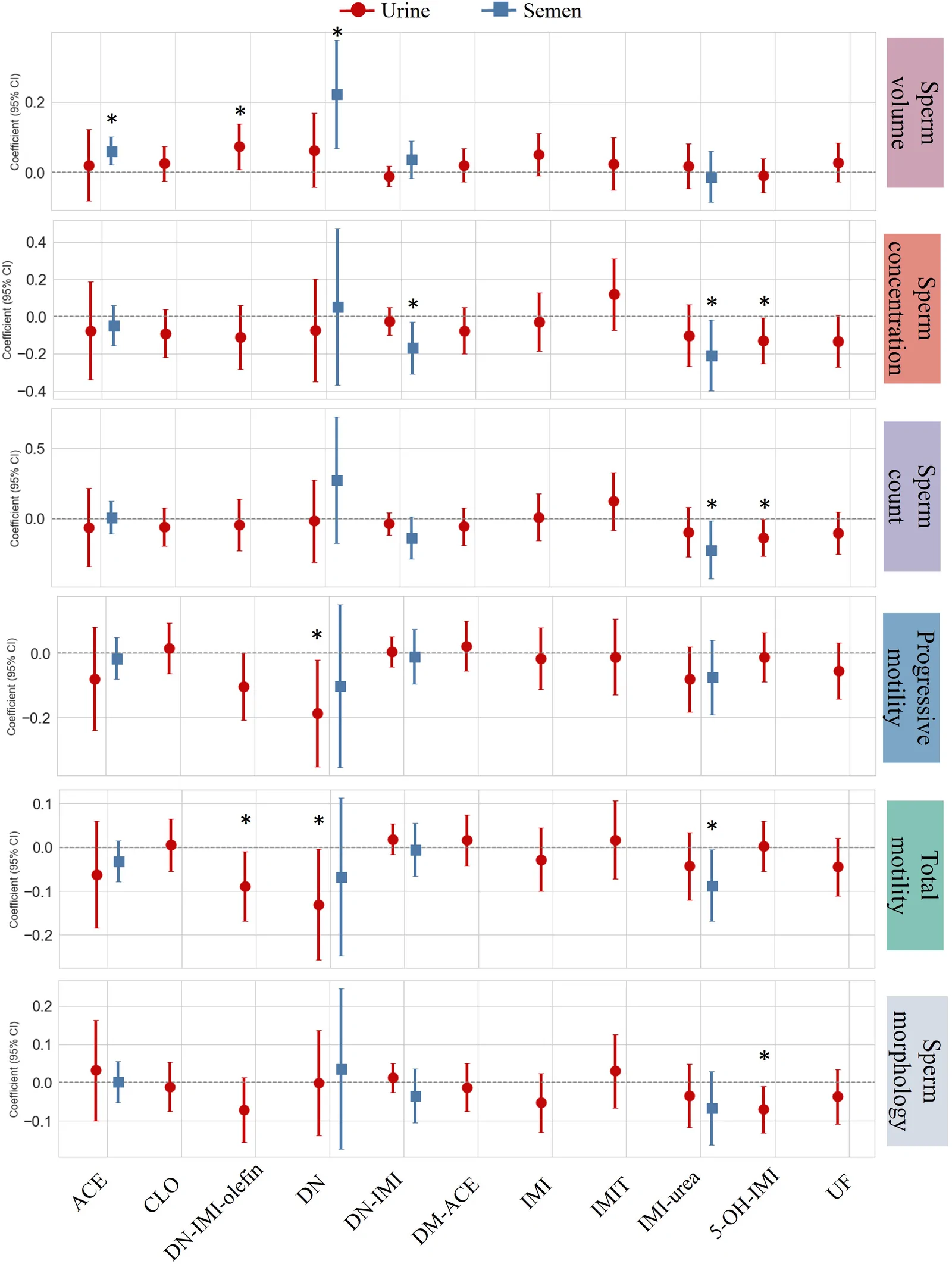
Associations between individual NEO concentrations in urine and semen and semen quality parameters. Multivariable linear regression models were used to estimate the associations between individual NEO concentrations in urine and semen and semen quality parameters. Points represent adjusted β coefficients, and error bars indicate 95% confidence intervals (CIs). All models were adjusted for relevant covariates. Asterisks (*) indicate statistically significant associations (*P* < 0.05). Models were adjusted for age, body mass index (BMI), education level, smoking status, season of sample collection, abstinence duration, and occupational exposure likelihood.

In analyses based on seminal NEOs levels, DN (β = 0.22, 95% CI, 0.07 to 0.38, *P* = 0.005) and ACE (β = 0.06, 95% CI, 0.02 to 0.10, *P* = 0.004) were positively associated with semen volume. DN-IMI was negatively associated with sperm concentration (β = –0.17, 95% CI, –0.31 to –0.03, *P* = 0.018). IMI-urea levels were inversely associated with sperm concentration (β = –0.21, 95% CI, –0.40 to –0.02, *P* = 0.029), total sperm count (β = –0.22, 95% CI, –0.43 to –0.02, *P* = 0.033), and total motility (β = –0.09, 95% CI, –0.17 to –0.01, *P* = 0.037). No statistically significant associations were observed for other compounds. Similar exposure–response trends were further identified in the quartile-based trend analyses (**Supplementary Figure S5**).

As shown in **Supplementary Figure S6**; **Supplementary Figure S7**, no significant exposure–response patterns or nonlinear associations were observed between NEOs in semen or urine and semen quality parameters, except for a few specific exposures. Notably, DN-IMI in semen was significantly associated with sperm concentration and total sperm count, and IMI-urea was associated with semen volume. In urine, IMI was significantly associated with total sperm count, and DM-ACE showed a significant nonlinear association with the percentage of morphologically normal sperm (*P* for nonlinear < 0.05). Stratified analyses by age revealed age-related differences in the observed associations. Specifically, the inverse associations of urinary DN with sperm concentration, DM-ACE and DN with total sperm count were significantly stronger among men younger than 35 years compared to those aged 35 years and above (*P* < 0.05). In contrast, the negative association between urinary IMI-urea and progressive motility was more pronounced in the older age group (≥ 35 years). No statistically significant effect modification by age was observed for the associations between seminal NEO concentrations and semen quality parameters (**Supplementary Table S5**).

### 3.4 Joint effects of NEO mixtures on semen quality

BKMR models were used to assess the associations between urinary and seminal NEO mixtures and semen quality parameters (**Figure 2**). After adjusting for potential confounders, seminal NEO mixtures were significantly associated with lower sperm concentration. The joint association of the mixtures remained statistically significant when all NEO levels were set either below or above the 50th percentile, compared with when all exposures were fixed at their medians; this pattern suggests a dose-dependent exposure–response pattern in which increasing the overall NEO mixture from low to high levels progressively reduces predicted sperm concentration. Analysis of PIPs identified DN-IMI as the most important individual contributor to the mixture–sperm concentration association (PIP = 0.913, indicating a >91% posterior probability that DN-IMI independently contributes to the mixture effect on sperm concentration) (**Supplementary Table S6**). In contrast, a positive association was observed between the seminal mixtures and semen volume, driven largely by ACE (PIP = 0.702, suggesting a moderately high probability that ACE contributes to this association). No statistically significant associations were found between the mixtures and sperm count, progressive motility, total motility, or morphology, although the overall trends were negative. For the urinary NEO mixtures, associations with semen quality parameters were not statistically significant, but the estimated directions of associations were generally consistent with those observed for seminal exposures.

**Figure 2:**
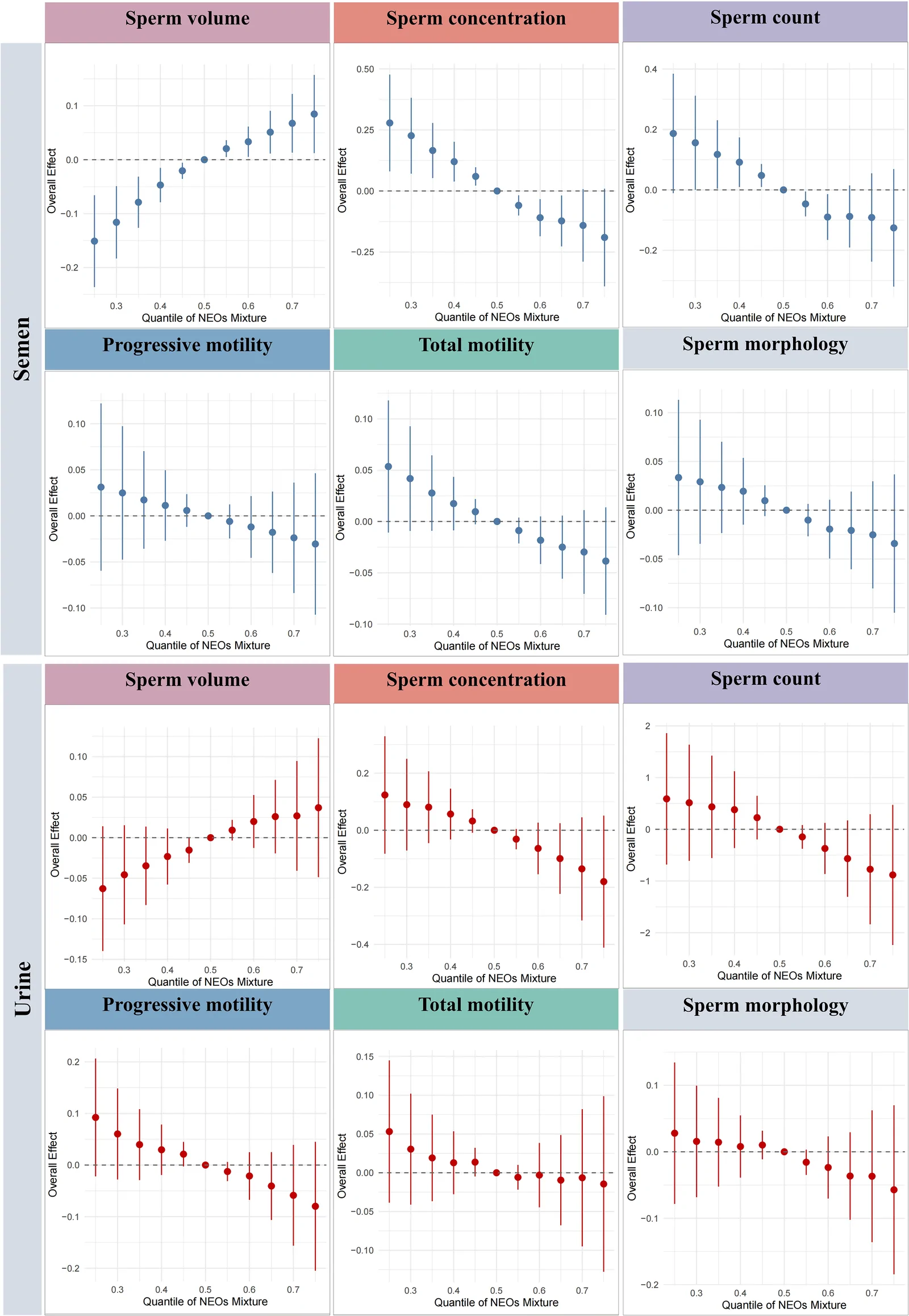
Overall mixture effects of NEOs on semen quality estimated by Bayesian kernel machine regression (BKMR). BKMR was used to estimate the overall effect of NEO mixtures on semen quality parameters. The overall mixture effect represents the change in each semen outcome when all NEO concentrations are simultaneously set to specific percentiles of their joint distribution, compared with when all exposures are fixed at their median levels (50th percentile). The x-axis indicates the percentiles (quantiles) of the joint exposure distribution (from the 25th to the 75th percentile), and the y-axis represents the estimated change in the outcome. Points indicate posterior mean estimates, and vertical bars represent 95% confidence intervals (CIs). The horizontal dashed line at zero indicates no association; associations are considered statistically significant when the 95% CIs does not include zero. Models were adjusted for age, body mass index (BMI), education level, smoking status, season of sample collection, abstinence duration, and occupational exposure likelihood.

Consistent with the BKMR findings, the QGC analyses showed that seminal NEO mixtures were significantly associated with lower sperm concentration, corresponding to an estimated %Δ = –27% (95% CI: –43% to –7%) per quartile increase in exposure; in practical terms, this means that a simultaneous increase in all seminal NEO concentrations from one quartile to the next was associated with an estimated 27% decrease in sperm concentration. Regarding the contribution of individual components, DN-IMI (weight = 0.407, meaning that DN-IMI contributed approximately 41% of the negative mixture weight) was the major contributor to this observed negative association, consistent with its high PIP in the BKMR analysis and reinforcing the convergent evidence from both mixture approaches. Seminal NEO mixtures were also positively associated with semen volume, with an estimated %Δ = 10% (95% CI: 1% to 22%). This positive mixture association was predominantly driven by ACE, which accounted for 82.0% of the positive weights, indicating that ACE was the primary driver of the observed increase in semen volume within the mixture framework. No significant associations were observed for sperm count, progressive motility, total motility, or morphology (**Figure 3**). For urinary NEO mixtures, none of the associations reached statistical significance; however, the directions of associations were generally consistent with those observed for seminal NEO mixtures.

**Figure 3:**
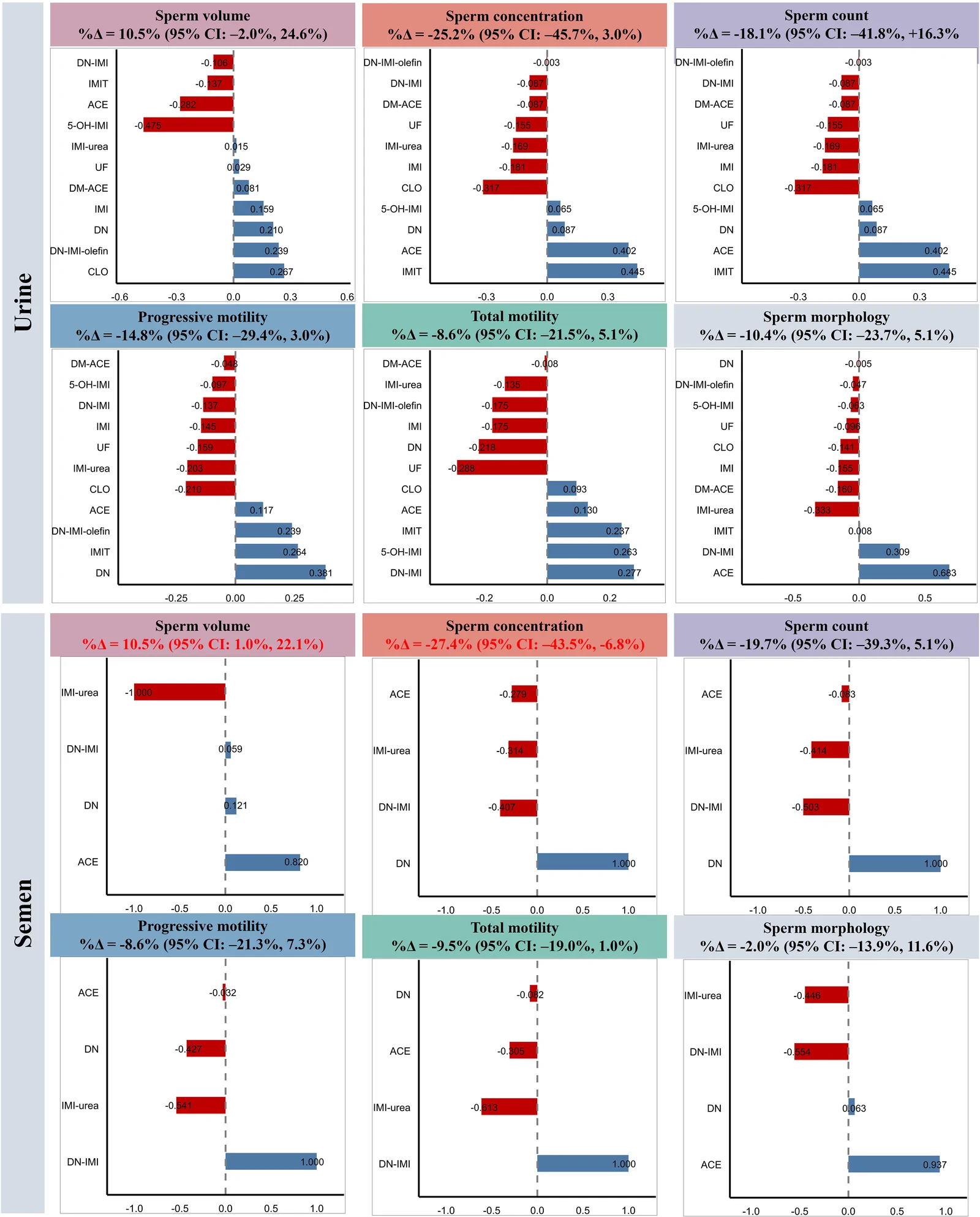
Quantile-based g-computation (QGC) analyses assess the joint associations between semen quality parameters and NEO mixtures in urine and in semen. Effect estimates are presented as percent changes (%Δ) with 95% confidence intervals (CI), representing the relative change in each semen parameter per one-quartile increase in NEO mixture concentrations. Models were adjusted for age, body mass index (BMI), education level, smoking status, season of sample collection, abstinence duration, and occupational exposure likelihood.

### 3.5 Sensitivity analyses

Sensitivity analyses confirmed the robustness of the primary findings. Specifically, restricting analyses to paired urine–semen samples, standardizing ln-transformed NEO concentrations using z-scores, additionally adjusting for semen volume, or re-imputing values below the LOD using the LOD/√2 method yielded results that were materially unchanged from the primary analyses (**Supplementary Tables S7–S10**).

## 4 Discussion

Using paired semen and urine samples from the same individuals, we simultaneously characterized NEOs and m-NEOs across both matrices—an approach that enabled the assessment of matrix-specific differences in detection patterns and concentrations. Eleven compounds were detected at high frequencies (>75%) in urine compared with only four in semen. Joint mixture analysis further showed that seminal NEO exposure was significantly associated with lower sperm concentration and higher semen volume, with estimated changes of –27% and 10%, respectively, per quartile increase in exposure.

The higher detection frequencies and concentrations in urine were consistent with findings for other environmental chemicals, such as phenols, phthalate metabolites, and parabens (35, 36). The higher detection frequencies and median concentrations in urine may be explained by two factors: first, m-NEOs are mainly excreted in urine following absorption and metabolism (18), rather than accumulating in reproductive tissues; second, the blood–testis barrier restricts the entry of environmental chemicals into the male reproductive tract (19). Consistent with previous research, we observed that the concentrations of m-NEOs in both semen and urine were generally higher than those of their parent compounds. For instance, Jia et al. reported that metabolites such as 5-OH-IMI and olefin-imidacloprid showed concentrations several orders of magnitude higher than IMI (37), while human biomonitoring data indicate that DM-ACE typically exhibits higher detection frequencies and concentrations than its parent ACE (38). Notably, although ACE showed a marginally higher urinary detection frequency than DM-ACE (100.00% vs. 95.53%), median urinary DM-ACE concentrations were approximately 6-fold higher than those of ACE (0.57 vs. 0.09 ng/mL), consistent with the metabolite-parent relationship described above. This pattern indicates that the small difference in detection frequency reflects sample-level variability near the LOD rather than analytical interference or signal misassignment, particularly given that MRM-based detection with retention-time matching against authentic standards was used throughout the analysis. These findings underscore that assessments based solely on parent compounds may underestimate total NEO exposure. The metabolic pathways of NEOs in humans are increasingly understood. Harada et al. demonstrated that IMI undergoes hepatic metabolism to yield 5-OH-IMI, IMI-urea, and DN-IMI (39), while Wrobel et al. identified DM-ACE as a primary human metabolite of ACE (40, 41). However, not all detected compounds necessarily originate from internal metabolism; some may result from environmental degradation or direct ingestion of preformed metabolites in food or water (42). Future studies employing controlled exposure protocols or animal models are needed to fully elucidate the metabolic fate of NEOs in humans.

Importantly, the matrix-specific exposure patterns observed in this study highlight the complementary value of seminal plasma measurements. Our previous study using paired semen and urine samples demonstrated distinct distribution patterns of NEOs and their metabolites between the two matrices and identified associations between NEO exposure profiles and male reproductive hormone levels, supporting the potential relevance of semen as a reproductive exposure matrix (20). The present study extends these findings by specifically investigating associations between NEO exposure profiles and semen quality parameters using single-pollutant and mixture-based approaches. While urinary m-NEO concentrations primarily reflect recent systemic exposure, absorption, metabolism, and renal elimination, seminal plasma represents the local extracellular environment surrounding spermatozoa and may provide additional information on reproductive tract exposure (21). Differences in toxicokinetic processes, including tissue distribution, protein binding, and selective transport across biological barriers such as the blood–testis barrier, may result in exposure profiles in seminal plasma that cannot be fully reflected by urinary biomarkers alone (21, 43). Therefore, seminal plasma may provide additional biologically relevant information for investigating potential reproductive health effects, particularly for environmental chemicals that may influence sperm development and function at low exposure levels (13).

In the single-pollutant models, metabolites of IMI showed the most consistent associations with altered semen quality. Urinary 5-OH-IMI was inversely associated with sperm concentration, total sperm count, and normal morphology, while in semen, both DN-IMI and IMI-urea were associated with lower sperm concentration. IMI-urea additionally showed negative associations with total sperm count and total motility. These converging signals suggest that IMI metabolites may contribute to the observed associations with semen quality, consistent with evidence that DN-IMI exhibits high mammalian toxicity (15, 16) and that IMI exposure has been reported to affect sperm quality, reproductive hormones, and testicular histology in rodents (13). For dinotefuran (DNT), its major metabolite DN demonstrated negative associations in urine, where higher DN concentrations were linked to lower progressive and total motility. Although DN is a key degradation and metabolic product of DNT, its toxicological characterization in mammals is extremely limited (44). Nevertheless, caution is required when extrapolating findings from experimental animal studies to human populations. In toxicological experiments, rodents are typically exposed to relatively high doses of NEOs under controlled conditions for defined exposure periods, whereas human populations are generally subject to chronic low-level exposure through dietary and environmental sources. In addition, species-specific differences in xenobiotic metabolism may influence internal exposure profiles. Imidacloprid is metabolized through oxidative and nitroreduction pathways mediated by cytochrome P450 enzymes to produce metabolites such as desnitro-imidacloprid (DN-IMI) and imidacloprid-urea, and the relative formation and clearance of these metabolites can vary between rodents and humans (45–47). These differences in exposure dose, duration, and metabolic processing may partly explain variations in the magnitude or pattern of observed reproductive associations across species.

Age-stratified analyses revealed that the inverse associations between urinary DN and sperm concentration, and between DN-IMI-olefin and total sperm count, were more pronounced in participants younger than 35 years compared to those aged 35 and above. In contrast, the negative association between urinary IMI-urea and progressive motility was stronger in the older age group. One study reported that serum IMI levels appeared to increase linearly with age (9). Previous studies have not found significant age-related differences in urine or serum NEO concentrations (16, 48–50). Given the reduced sample size after stratification, these age-specific associations should be interpreted as exploratory and hypothesis-generating findings. Further studies with larger sample sizes and longitudinal designs are needed to confirm whether age modifies the relationship between NEO exposure, metabolism, and male reproductive outcomes.

The most clinically significant finding emerged from mixture analyses using BKMR and QGC models. Seminal NEO mixtures were significantly associated with lower sperm concentration, with QGC analysis estimating a 27% reduction in sperm concentration (95% CI: –43% to –7%) per quartile increase in exposure. This magnitude of association may be clinically relevant, considering that the 2021 WHO lower reference limit for sperm concentration is 16 million/mL, representing the 5th percentile among men who achieved natural conception within 12 months (51, 52). For men with borderline baseline sperm concentrations (e.g., 20–25 million/mL), a 27% reduction associated with higher exposure levels could correspond to values below this diagnostic threshold, potentially affecting fertility classification. Such findings are consistent with the nonlinear clinical impact of changes occurring near diagnostic cutoffs, where relatively small declines in semen quality may substantially alter fertility classification and clinical management. Moreover, sperm concentration is closely correlated with total motile sperm count (TMSC), an important predictor of natural conception and a key parameter in assisted reproductive decision-making. TMSC values below approximately 10 million are associated with reduced fecundity and an increased likelihood of requiring assisted reproductive technologies (53, 54). Therefore, the lower sperm concentration associated with seminal NEO mixtures may also translate into clinically meaningful decreases in TMSC, particularly among men with already compromised semen quality. Collectively, these findings suggest that even modest population-level differences in sperm concentration associated with environmental NEO exposure may have important reproductive implications in susceptible individuals.

Interestingly, we also observed a positive association between seminal NEO concentrations and semen volume, with an approximately 10% increase per quartile of exposure, and similar associations were observed for individual compounds (DN, ACE, DN-IMI-olefin). Although this direction differs from the negative association observed for sperm concentration, semen volume should be interpreted cautiously because it primarily reflects secretory activity of the accessory sex glands (particularly the seminal vesicles and prostate) and is influenced by physiological and procedural factors such as abstinence interval, rather than directly reflecting spermatogenesis or intrinsic sperm function (55). Similar positive associations between endocrine-disrupting chemical biomarkers and semen volume have been reported in some human studies, although findings across semen parameters remain inconsistent (56). One possible explanation is that endocrine-disrupting chemicals may influence accessory gland function through disruption of androgen signaling, oxidative stress, or inflammatory pathways without necessarily being associated with improved spermatogenesis or sperm function (57). In addition, nonmonotonic dose–response relationships at low exposure levels may contribute to unexpected positive associations within restricted exposure ranges (58). However, given the limited epidemiological evidence regarding NEO exposure and semen volume and the cross-sectional design of this study, these findings require cautious interpretation and further validation in longitudinal studies incorporating biomarkers of accessory gland function.

Our study has several significant advantages. By analyzing both semen and urine samples from the same individuals, we were able to characterize NEO and m-NEO concentrations across systemic and reproductive matrices. These results reinforce the utility of urine as a practical and noninvasive matrix for population-level biomonitoring of NEO exposure, while highlighting the complementary value of semen measurements for reproductive risk assessment. Our findings provide preliminary evidence that exposure to NEOs and m-NEOs may adversely affect male reproductive health, as reflected in altered sperm quality. These results underscore the need for greater caution regarding NEO exposure and support the exploration of safer pest management alternatives that protect both agricultural productivity and human health. Ongoing research and regulatory efforts are crucial for a deeper understanding and mitigation of the potential risks posed by NEOs.

Nonetheless, several limitations should be acknowledged. First, participants were recruited from a fertility clinic rather than the general population, which may limit generalizability. Although semen parameters in our cohort were comparable to population-based estimates, the scarcity of studies on NEOs in male populations prevents determining whether clinic attendees differ in exposure levels or susceptibility. Future studies should include population-based sampling to enhance external validity. Multi-center designs involving both community-based participants and clinic populations would allow direct comparison of exposure distributions and association estimates. Furthermore, stratified analyses or weighting approaches could be applied to account for potential selection bias. Second, residual confounding cannot be excluded because information on diet, physical activity, and other environmental pollutants was unavailable. Spermatogenesis is highly sensitive to lifestyle and environmental factors, including nutrient intake (e.g., creatine, myo-inositol) (59), bioactive compounds in certain foods (60), exercise and sedentary behavior (61, 62), and exposures to chemicals released from plastics or microplastics (63). These unconsidered variables may potentially influence the observed associations in our study. Future studies should incorporate more comprehensive assessments of lifestyle, dietary patterns, and co-exposure to environmental contaminants to better control for potential residual confounding. Third, the cross-sectional design limits causal inference. Longitudinal cohort studies with repeated exposure measurements, together with the integration of mechanistic biomarkers, are needed to strengthen causal inference and to further elucidate the underlying biological pathways. Finally, like most prior studies, we assessed only a subset of individual chemicals, whereas humans are co-exposed to complex mixtures that may exert additive or synergistic effects not captured by single-pollutant analyses. Future studies incorporating multipollutant models, mixture analysis approaches, or exposome-based frameworks are warranted to better characterize the combined associations of complex environmental exposures with male reproductive health.

## 5 Conclusion

This study provides a direct comparison of NEO concentrations in semen and urine samples and their associations with semen quality in men. The target chemicals were detected at high frequencies in both matrices, suggesting widespread human exposure. These results provide preliminary evidence that exposure to NEOs and m-NEOs may be associated with alterations in male reproductive health indicators, as reflected by altered semen quality. Our findings underscore the role of semen as a biologically relevant matrix for evaluating reproductive exposure to NEOs, and highlight the value of urine as a more accessible alternative when semen samples are unavailable. To further clarify the reproductive implications of NEO exposure, well-designed prospective cohort studies are essential to validate these associations, minimize potential bias, and elucidate underlying biological pathways.

## Data Availability

The original contributions presented in the study are included in the article/**Supplementary Material**. Further inquiries can be directed to the corresponding authors.
