# Supplementary material for: Simultaneous semen and urine biomonitoring of neonicotinoids and associations with semen quality in men
Source: Front Endocrinol (Lausanne). 2026 Sep 28;17:1871388. doi: 10.3389/fendo.2026.1871388 (PMC13654715; doi:10.3389/fendo.2026.1871388)
Supplement: Supplementary file 1 [file SupplementaryFile1.docx]

Supplementary Text S1. Directed acyclic graph describing the hypothesized causal structure for the association between neonicotinoid insecticide (NEO) exposure and semen quality parameters

**Overview**

We constructed a directed acyclic graph (DAG) to identify a minimally sufficient set of confounders for estimating the association between NEO exposure and semen quality parameters. The DAG was informed by toxicological evidence suggesting reproductive effects of NEOs and by epidemiologic knowledge on determinants of exposure and semen quality.

**S1.1 Exposure–outcome relation**

NEO exposure was specified as the primary exposure and semen quality parameters as outcomes. Experimental and human-health–relevant evidence indicates that neonicotinoids can disrupt male reproductive function and sperm quality, supporting a plausible causal pathway from NEO exposure to altered semen parameters (1)(2).

**S1.2 Confounders affecting both NEO exposure and semen quality**

**Age.**

Age was assumed to influence internal NEO exposure through cumulative environmental/occupational contact patterns and to influence semen quality via reproductive aging (3)(4).

**Body mass index (BMI).**

BMI was treated as a confounder because adiposity can affect toxicant distribution/metabolism and biomarker levels, and abnormal BMI is consistently associated with poorer semen parameters (3)(5).

**Education level.**

Education level was included as a confounder representing socioeconomic position that shapes dietary patterns, residential environment, and occupation, thereby influencing NEO exposure opportunities, while also being associated with reproductive health and semen quality (3)(6).

**Smoking status.**

Smoking status was assumed to affect NEO exposure because smoking-related behaviors and environments may co-vary with pesticide contact and other exposure sources, and smoking is a well-established risk factor for reduced sperm concentration, motility, and morphology (7).

**Occupational exposure likelihood.**

Occupational exposure likelihood (high vs. low) was assumed to directly increase NEO exposure via workplace use/handling of pesticides and to be independently associated with semen quality through occupational hazards and co-exposures; thus, it is a confounder rather than a mediator (8).

**Season of sample collection.**

Season was treated as a confounder because (i) neonicotinoid exposure biomarkers show temporal/seasonal variability related to pesticide application and environmental levels, and (ii) semen parameters demonstrate circannual patterns associated with temperature/photoperiod and seasonal environmental conditions (9)(10).

**Abstinence duration.**

Abstinence duration is a direct determinant of semen volume, sperm concentration, and motility, and may be correlated with age and lifestyle; therefore abstinence duration was included to close potential backdoor paths and reduce measurement-related bias in semen parameters (11)(12).

**S1.3 Assumed relations among covariates**

We assumed that education level influences smoking status and occupational exposure likelihood (education → smoking; education → occupation), reflecting well-documented socioeconomic gradients in tobacco use and job types (13)(14). We also assumed that age may influence abstinence duration (age → abstinence) through behavioral and reproductive-life–course factors commonly observed in andrology cohorts (15).

**S1.4 Final adjustment set**

Under these assumptions, the minimal sufficient adjustment set to block all backdoor paths between NEO exposure and semen quality comprised: age (years), BMI (kg/m²), education level (below bachelor’s degree vs. bachelor’s degree or above), smoking status (yes or no), season of sample collection (spring, summer, or autumn), abstinence duration (<3, 3–5, or >5 days), and occupational exposure likelihood (low vs. high). These variables were therefore included in the primary regression models.

Reference

1.	Irfan SS, Sanchez VG, Bloom MS, Chin HB, Espina V, Krall JR, et al. Reproductive risk of Neonicotinoids: A review of male rodent studies. Environ Res. 2025 Dec 1;286(Pt 3):122903.

2.	Oladosu JI, Flaws JA. The impact of neonicotinoid pesticides on reproductive health. Toxicol Sci. 2024 Oct 26;203(2):131–46.

3.	Zhang D, Lu S. Human exposure to neonicotinoids and the associated health risks: A review. Environ Int. 2022 May 1;163:107201.

4.	Johnson SL, Dunleavy J, Gemmell NJ, Nakagawa S. Consistent age-dependent declines in human semen quality: A systematic review and meta-analysis. Ageing Res Rev. 2015 Jan 1;19:22–33.

5.	Li Y, Lin Y, Ou C, Xu R, Liu T, Zhong Z, et al. Association between body mass index and semen quality: a systematic review and meta-analysis. Int J Obes 2005. 2024 Oct;48(10):1383–401.

6.	Liu W, Zhao L, Yang X, Gu L, Liu W, Zhu H. Exposure to ambient particulate matter and semen quality: the modifying role of socioeconomic status and lifestyle factors in chinese men. BMC Public Health. 2025 Oct 1;25(1):3263.

7.	Sharma R, Harlev A, Agarwal A, Esteves SC. Cigarette Smoking and Semen Quality: A New Meta-analysis Examining the Effect of the 2010 World Health Organization Laboratory Methods for the Examination of Human Semen. Eur Urol. 2016 Oct;70(4):635–45.

8.	Li J, Zhang H, Zhao K, Han C, Li C, Fang L, et al. An occupational health assessment of dinotefuran exposure in greenhouse vegetable workers: Metabolomic profiling and toxicokinetic analysis. J Hazard Mater. 2025 Jul 5;491:137989.

9.	Li F, Lin X, Liu J. Variability of urinary biomarkers of neonicotinoid insecticides in Chinese population: Implications for human exposure assessment. Chemosphere. 2022 Nov;307(Pt 1):135705.

10.	Nazem TG, Sekhon L, Lee J, Briton-Jones C, Bar-Chama N, Copperman AB. Seasonal variation of semen quality from over 100,000 analyzed samples. Fertil Steril. 2018 Sep 1;110(4):e169–70.

11.	World Health Organization. WHO laboratory manual for the examination and processing of human semen [Internet]. World Health Organization; 2021 [cited 2025 Nov 26]. Available from: https://www.who.int/publications/b/59107

12.	Björndahl L, Brown JK. The sixth edition of the WHO Laboratory Manual for the Examination and Processing of Human Semen: ensuring quality and standardization in basic examination of human ejaculates. Fertil Steril. 2022 Feb 1;117(2):246–51.

13.	Huang MZ, Liu TY, Zhang ZM, Song F, Chen T. Trends in the distribution of socioeconomic inequalities in smoking and cessation: evidence among adults aged 18 ~ 59 from China Family Panel Studies data. Int J Equity Health. 2023 May 11;22(1):86.

14.	Droomers M, Huang X, Fu W, Yang Y, Li H, Zheng P. Educational disparities in the intention to quit smoking among male smokers in China: a cross-sectional survey on the explanations provided by the theory of planned behaviour. BMJ Open. 2016 Oct 7;6(10):e011058.

15.	Castellini C, Cordeschi G, Tienforti D, Barbonetti A. Relationship between male aging and semen quality: a retrospective study on over 2500 men. Arch Gynecol Obstet. 2024 Jun;309(6):2843–52.
