# Supplementary material for: Simultaneous semen and urine biomonitoring of neonicotinoids and associations with semen quality in men
Source: Front Endocrinol (Lausanne). 2026 Sep 28;17:1871388. doi: 10.3389/fendo.2026.1871388 (PMC13654715; doi:10.3389/fendo.2026.1871388)
Supplement: Supplementary file 2 [file SupplementaryFile2.docx]

**Supplementary Methods**

**S1. Detailed UPLC-MS/MS chromatographic and detection conditions**

Chromatographic separation was carried out on an ACQUITY UPLC BEH C18 column (100 mm × 2.1 mm, 1.7 µm) at room temperature. The mobile phases consisted of 0.1% formic acid in water (A) and acetonitrile (B). A total of 5 µL of each sample was injected, and compounds were eluted at a constant flow rate of 0.30 mL/min using the following gradient: 0–1 min, 98% A / 2% B; 1–3.5 min, 0% A / 100% B; 3.5–5.5 min, 0% A / 100% B; 5.5–6.5 min, 98% A / 2% B; and 6.5–7 min, 98% A / 2% B for re-equilibration. The system dwell volume was approximately 300 µL, corresponding to a calculated dwell time of ~1.0 min at this flow rate (300 µL / 0.30 mL/min). The initial 1-min isocratic hold at 98% A (0–1 min) was designed to accommodate this dwell time, ensuring that the target analytes, which elute at approximately 2–3 min, are resolved under the intended post-dwell gradient conditions rather than during the system's delay volume. Multiple reaction monitoring (MRM) in positive electrospray ionization mode was conducted. Detailed quantification parameters, including precursor ions, product ions, cone voltages, and collision energies for each analyte, are provided in **Table S2**.

**S2. Detailed quality assurance and quality control (QA/QC) procedures**

To account for background contamination, ten procedural blanks were analyzed and used to subtract baseline levels of NEOs. Instrumental blanks were analyzed every 20 samples to monitor potential contamination. Analyte identification was based on retention time, quantification and confirmation ions.

Quantification was performed using ten-point calibration curves (0–200 ng/mL), which showed excellent linearity for all analytes, with correlation coefficients (R²) ranging from 0.991 to 1.000 in both matrices. The limit of detection (LOD) and limits of quantification (LOQ) were based on the lowest concentration of the spiked levels with a signal-to-noise (S/N) ratio of 3:1 and 10:1, respectively (1). Method detection limits (MDLs) and method quantification limits (MQLs) were set as 3 and 10 times, respectively, the standard deviation of target analyte levels in procedural blanks (2). The LODs for the analytes ranged from 0.003 to 0.089 ng/mL in urine and 0.021 to 1.087 ng/mL in semen.

Accuracy and precision of the analytical method were evaluated through spiked recovery experiments. For urine, mean recoveries ranged from 87.25% (Desmethyl-acetamiprid, DM-ACE) to 110.00% (Imidacloprid-urea, IMI-urea; Imidaclothiz, IMIT), with RSDs between 7.80% (Clothianidin, CLO) and 21.65% (5-Hydroxy-imidacloprid, 5-OH-IMI). For semen, recoveries ranged from 75.99% (CLO) to 129.85% (5-OH-IMI), with RSDs from 5.02% (UF) to 17.58% (Desmethyl-thiamethoxam, DM-THM).

Matrix effects (ME) were assessed using the slope-ratio method (3), calculated as the ratio between the slopes of matrix-matched and solvent-based calibration curves. ME values ranged from 0.14 (DM-THM) to 2.27 (Desnitro-dinotefuran, DN) in urine and 1.02 (Desnitro-imidacloprid, DN-IMI) to 3.59 (DM-ACE) in semen, indicating mild ion suppression to strong enhancement depending on the analyte. Isotope-labeled internal standards effectively compensated for these matrix-induced variations.

For analytes with concentrations below the LOD, values were treated as non-detectable (ND) and imputed as follows: (i) LOD/2 for compounds with detection frequencies (DFs) > 50%, or (ii) f × LOD for those with DFs < 50%, where f represents the proportion of detectable samples (4, 5). Urinary NEO concentrations were further corrected for dilution using specific gravity (SG) according to the formula: *C_SG_* = *C* × [(*SGmean* – 1)/(*SG* – 1)], where *C_SG_* is the SG-adjusted concentration, *C* is the measured concentration, *SG* is the specific gravity of the individual sample, and *SGmean* is the arithmetic mean of *SG* across the study population (31). SG-corrected concentrations were used for all subsequent analyses.

**S3. Detailed BKMR and QGC model specifications**

**Bayesian kernel machine regression (BKMR)**

BKMR is a semi-parametric method that flexibly models potentially non-linear and interactive effects among multiple exposures. Prior to modeling, NEO concentrations were natural log-transformed and standardized using z-scores to improve model stability and comparability across exposures. All models were adjusted for relevant covariates and fitted using a Markov Chain Monte Carlo (MCMC) algorithm with 10,000 iterations (6). The overall mixture effect was estimated by comparing predicted semen quality outcomes when all NEOs were simultaneously set at different percentiles (from the 25th to the 75th percentile in 5-percentile increments), relative to the median level of all exposures. In practical terms, this approach estimates how semen quality outcomes would be expected to change if the entire NEO mixture increased together from a lower to a higher exposure level, compared with everyone being at the median (50th percentile) exposure. Although exposure–response functions for individual NEOs were also estimated by varying each exposure while holding the remaining exposures at their median values, these results were not presented, as similar exposure–response patterns were already captured using restricted cubic spline models.

To further assess the relative importance of individual exposures within the mixture, posterior inclusion probabilities (PIPs) were calculated based on the MCMC samples. PIP reflects the probability that a given exposure is included in the model and serves as an indicator of variable importance within the mixture. In simpler terms, PIP can be understood as a measure of how confident the model is that a given NEO meaningfully contributes to the overall mixture effect on semen quality. PIP values range from 0 to 1, with values closer to 1 indicating a higher likelihood that the exposure is an important contributor to the mixture effect, whereas values closer to 0 suggest limited or negligible contribution. As a general reference, exposures with PIP > 0.5 are often considered relatively more important within the mixture, although this threshold is not a strict cutoff and should be interpreted alongside the exposure–response patterns. Model convergence was evaluated using Gelman–Rubin diagnostics (R-hat) and visual inspection of trace plots. R-hat values for all parameters ranged from 1.00 to 1.08, indicating adequate convergence (**Table S3; Figure S3**).

**Quantile-based g-computation (QGC)**

QGC is a parametric extension of g-computation based on generalized linear models, designed to estimate the joint effect of chemical mixtures (7). QGC allows mixture components to exhibit associations in different directions. A Gaussian link function was specified. The number of quantiles was set to four (q = 4) to ensure an adequate number of observations within each exposure category given the sample size (n = 173), thereby improving estimation stability while maintaining sufficient resolution to detect potential dose–response relationships. Bootstrap resampling (1,000 iterations) was used to derive regression coefficients and 95% confidence intervals, representing the change in semen parameters per one-quartile increase in the NEO mixtures index.

In addition, weights for individual components were estimated to quantify their relative contributions to the overall mixture effect. These weights can be interpreted as an index of each chemical's relative contribution to the overall mixture association, conceptually similar to standardized regression coefficients. Positive and negative weights indicate the direction of a component's contribution to the overall mixture effect (positive or negative direction, respectively), and within each direction, weights sum to 1, with larger weights indicating a proportionally greater contribution to the mixture effect in that direction. For example, if a given exposure has a positive weight of 0.6, it indicates that this chemical accounts for approximately 60% of the overall positive mixture effect, making it a primary driver among the exposures associated with an increase in the outcome. This allows readers to readily identify which specific NEOs contribute most strongly to the observed mixture effect, in either the positive or negative direction.

**Sensitivity analyses**

Several sensitivity analyses were conducted to assess the robustness of the findings. These analyses included: (1) restricting analyses to participants with paired urine–semen samples; (2) standardizing ln-transformed NEO concentrations using z-scores; (3) additionally adjusting for semen volume in models evaluating seminal NEOs; and (4) re-imputing values below the LOD using the LOD/√2 method to evaluate the impact of alternative imputation approaches.

**Reference**

1. Z. Cheng, et al., A target screening method for detection of organic pollutants in fruits and vegetables by atmospheric pressure gas chromatography quadrupole-time-of-flight mass spectrometry combined with informatics platform. *J. Chromatogr. A* **1577**, 82-91 (2018).

2. L. Zhao, et al., E-waste dismantling-related occupational and routine exposure to melamine and its derivatives: estimating exposure via dust ingestion and hand-to-mouth contact. *Environ. Int.* **165**, 107299 (2022).

3. B. K. Matuszewski, M. L. Constanzer, C. M. Chavez-Eng, Strategies for the assessment of matrix effect in quantitative bioanalytical methods based on hplc-ms/ms. *Anal. Chem.* **75**, 3019-3030 (2003).

4. S. Zhang, et al., Trans-placental transfer mechanisms of aromatic amine antioxidants (aas) and p-phenylenediamine quinones (ppd-qs): evidence from human gestation exposure and the rat uterine perfusion model. *Environ. Sci. Technol.* **58**, 21166-21176 (2024).

5. Y. Li, et al., Direct evidence on occurrence of emerging liquid crystal monomers in human serum from e-waste dismantling workers: implication for intake assessment. *Environ. Int.* **169**, 107535 (2022).

6. J. F. Bobb, et al., Bayesian kernel machine regression for estimating the health effects of multi-pollutant mixtures. *Biostatistics (Oxford, England)* **16**, 493-508 (2015).

7. A. P. Keil, et al., A quantile-based g-computation approach to addressing the effects of exposure mixtures. *Environ. Health Perspect.* **128**, 47004 (2020).
