# Supplementary material for: Simultaneous semen and urine biomonitoring of neonicotinoids and associations with semen quality in men
Source: Front Endocrinol (Lausanne). 2026 Sep 28;17:1871388. doi: 10.3389/fendo.2026.1871388 (PMC13654715; doi:10.3389/fendo.2026.1871388)
Supplement: Supplementary file 3 [file Table1.docx]

***Supporting Information***

**Simultaneous Semen and Urine Biomonitoring of Neonicotinoids and Associations with Semen Quality in Men**

Li-Jun Lei^a#^, Lu Zhang^b#^, Qing-Shuang Yang^b^, Zi-Jun Zhou^c^, Jiao-Jing Liu^a^, Fang-Di Dong^a^, Zhi-Wei Xue^a^, Ling-Yuan Wu^a^, Zhi-Peng Cheng^c^, Ping-Ping Wang^d^*, Yu-Bin Ding^a,b,e^*, Hui-Jun Yang^a^*

a Assisted Reproductive Center, Key Laboratory of Birth Regulation and Control Technology of National Health Commission of China, Shandong Provincial Key Medical and Health Discipline of Reproductive Medicine, Shandong Provincial Maternal and Child Health Care Hospital Affiliated to Qingdao University, Jinan, 250014, China

b Joint International Research Laboratory of Reproduction and Development of the Ministry of Education of China, School of Public Health, Chongqing Medical University, Chongqing 400016, China

c MOE Key Laboratory of Pollution Processes and Environmental Criteria, College of Environmental Science and Engineering, Nankai University, Tianjin, 300350, China

d National Engineering Research Center of Pesticide, College of Chemistry, Nankai University, Tianjin 300071, China

e Department of Pharmacology, Academician Workstation, Changsha Medical University, Changsha 410219, China

*** Corresponding authors:**

**Hui-Jun Yang**, E-mail: [lwyhj123@1](mailto:lwyhj123@1)63.com

**Yu-Bin Ding**, E-mail: [dingyb@cqmu.edu.cn](mailto:dingyb@cqmu.edu.cn)

**Ping-Ping Wang**, E-mail: wangpingping@nankai.edu.cn

\# These authors contributed equally.

* These authors jointly supervised this work.

Table S1. The detailed information, including full name, abbreviation, Chemical Abstracts Service Registry Number (CAS), formula, molecular weight (MW), structure**,** of 18 neonicotinoid insecticides (NEOs) and 5 isotopically labeled internal standards (ISs).

| **No.** | **Full Name** | **Abbreviation** | **CAS** | **Formula** | **Log KW^#^** | **MW** | **Structure** |
| --- | --- | --- | --- | --- | --- | --- | --- |
| 1 | Desnitro-dinotefuran | DN | 457614-32-3 | C₇H₁₅N₃O | / | 157.21 |  |
| 2 | Dinotefuran-urea | UF | 457614-34-5 | C₇H₁₄N₂O₂ | / | 158.2 |  |
| 3 | Descyano-acetamiprid | Descyno-ACE | 365441-66-3 | C_9_H_12_ClN_3_ | / | 199.76 |  |
| 4 | Desnitro-imidacloprid-olefin | DN-IMI-olefin | 187022-17-9 | C_9_H_9_ClN_4_ | 1.41 | 208.65 |  |
| 5 | Desnitro-imidacloprid | DN-IMI | 115970-17-7 | C_9_H_11_ClN_4_ | / | 210.67 |  |
| 6 | Dinotefuran | DNT | 165252-70-0 | C₇H₁₄N₄O₃ | -0.19 | 202.21 |  |
| 7 | Imidacloprid-urea | IMI-urea | 120868-66-8 | C_9_H_10_ClN_3_O | 0.64 | 211.65 |  |
| 8 | Thiamethoxam | THM | 153719-23-4 | C_8_H_10_ClN_5_O_3_S | 0.8 | 292.71 |  |
| 9 | 5-Hydroxy-imidacloprid | 5-OH-IMI | 155802-61-2 | C_9_H_10_ClN_5_O_3_ | -0.98 | 271.66 |  |
| 10 | Thiacloprid-amide | THCP-amide | 676228-91-4 | C_10_H_11_ClN_4_OS | 0.02 | 270.74 |  |
| 11 | Desmethyl-acetamiprid | DM-ACE | 190604-92-3 | C_9_H_9_ClN_4_ | 2.34 | 208.65 |  |
| 12 | Clothianidin | CLO | 210880-92-5 | C_6_H_8_ClN_5_O_2_S | 0.64 | 249.68 |  |
| 13 | Imidacloprid | IMI | 138261-41-3 | C_9_H_10_ClN_5_O_2_ | -0.41 | 255.66 |  |
| 14 | Acetamiprid | ACE | 135410-20-7 | C_10_H_11_ClN_4_ | 2.55 | 222.68 |  |
| 15 | Imidaclothiz | IMIT | 105843-36-5 | C_7_H_8_ClN_5_O_2_S | -0.23 | 261.69 |  |
| 16 | Desmethyl-thiamethoxam | DM-THM | 171103-04-1 | C_7_H_8_ClN_5_O_3_S | -0.93 | 277.69 |  |
| 17 | Flonicamid | FLO | 158062-67-0 | C₉H₆F₃N₃O | 0.5 | 229.16 |  |
| 18 | Thiacloprid | THCP | 111988-49-9 | C_10_H_9_ClN_4_S | 2.33 | 252.73 |  |
| 19 | Thiamethoxam-*d₄* | THM-*d₄* | 1331642-98-8 | C_8_H_6_D_4_ClN_5_O_3_S | / | 296.15 |  |
| 20 | Clothianidin-*d₃* | CLO-*d₃* | 1262776-24-8 | C_6_H_5_D_3_ClN_5_O_2_S | 0.64 | 253.00 |  |
| 21 | Imidacloprid-*d₄* | IMI-*d₄* | 1015855-75-0 | C₉H₆D₄ClN₅O₂ | -0.43 | 260.10 |  |
| 22 | Acetamiprid-*d₃* | ACE-*d₃* | 1353869-35-8 | C₁₀H₈D₃ClN₄ | / | 226.10 |  |
| 23 | Thiacloprid-(thiazolidinring-*d₄*) | THCP-*d₄* | 1793071-39-2 | C_10_D_4_H_5_ClN_4_S | / | 257.10 |  |

^#^: The log Kow values for the analytes were from ChemSpider (<http://www.chemspider.com/>).

Table S2. The detailed quantification information of 18 NEOs in human urine and semen samples.

| **Abbreviation** | **Quantification ion** | **Confirmation ion** | **RT (min)** | **Urine** | | | | | |  | **Semen** | | | | | |
| --- | --- | --- | --- | --- | --- | --- | --- | --- | --- | --- | --- | --- | --- | --- | --- | --- |
|  |  |  |  | **LOD (ng/mL)** | **LOQ (ng/mL)** | **R^2^** | **Recovery**  **(%)** | **RSD**  **(%)** | **ME** |  | **LOD (ng/mL)** | **LOQ (ng/mL)** | **R^2^** | **Recovery**  **(%)** | **RSD**  **(%)** | **ME** |
| DN ^a^ | 158.2>102.2 | 158.2>57.1 | 2.44 | 0.093 | 0.310 | 0.999 | 88.09 | 13.82 | 2.27 |  | 0.777 | 2.591 | 0.999 | 94.06 | 12.45 | 1.97 |
| UF ^a^ | 159.2>102.2 | 159.2>58.1 | 2.56 | 0.062 | 0.206 | 0.9999 | 89.32 | 16.73 | 0.83 |  | 0.292 | 0.973 | 0.999 | 102.29 | 5.02 | 2.08 |
| Descyno-ACE ^a^ | 198.2>126.1 | 198.2>90.1 | 2.63 | - | - | - | - | - | - |  | - | - | - | - | - | - |
| DN-IMI-olefin ^c^ | 209.5>126 | 209.5>90.1 | 2.68 | 0.023 | 0.077 | 0.999 | 87.42 | 14.23 | 1.58 |  | 0.050 | 0.166 | 0.999 | 99.27 | 6.79 | 1.57 |
| DN-IMI ^c^ | 211.1>126 | 211.1>84 | 2.69 | 0.003 | 0.010 | 0.999 | 97.33 | 13.83 | 0.85 |  | 0.021 | 0.071 | 0.9999 | 113.72 | 6.64 | 1.02 |
| DNT ^a^ | 203.1>129.1 | 203.1>157.2 | 2.73 | - | - | - | - | - | - |  | 1.087 | 3.623 | 1 | 87.10 | 12.76 | 1.42 |
| IMI-urea ^c^ | 212.1>128 | 212.1>99 | 2.97 | 0.025 | 0.084 | 0.999 | 110.00 | 17.54 | 1.18 |  | 0.313 | 1.042 | 0.991 | 127.02 | 6.98 | 2.88 |
| THM ^e^ | 295.1>214.1 | 295.1>184.1 | 3.00 | - | - | - | - | - | - |  | - | - | - | - | - | - |
| 5-OH-IMI ^c^ | 272.1>191.1 | 272.1>225.1 | 3.02 | 0.089 | 0.298 | 0.9999 | 97.08 | 21.65 | 0.93 |  | 0.794 | 2.645 | 0.997 | 129.85 | 8.90 | 2.65 |
| THCP-amide | 271.1>254.1 | 271.1>228.1 | 3.02 | - | - | - | - | - | - |  | - | - | - | - | - | - |
| DM-ACE ^a^ | 209.1>126 | 209.1>90.1 | 3.10 | 0.067 | 0.223 | 0.999 | 87.25 | 20.73 | 0.42 |  | 0.106 | 0.355 | 0.995 | 110.97 | 9.29 | 3.59 |
| CLO ^b^ | 250>168.9 | 250>131.9 | 3.12 | 0.016 | 0.053 | 0.999 | 108.00 | 7.80 | 1.03 |  | 0.163 | 0.542 | 0.999 | 75.99 | 12.79 | 1.64 |
| IMI ^c^ | 256.3>209.1 | 256.3>175.2 | 3.18 | 0.032 | 0.106 | 0.999 | 107.08 | 14.08 | 1.18 |  | 0.497 | 1.657 | 0.998 | 89.51 | 5.56 | 1.65 |
| ACE ^a^ | 223.2>126.1 | 223.2>56.1 | 3.21 | 0.025 | 0.083 | 1 | 105.58 | 15.92 | 1.09 |  | 0.027 | 0.091 | 0.9999 | 91.08 | 10.20 | 1.49 |
| IMIT ^c^ | 262>181 | 262>121.9 | 3.22 | 0.033 | 0.109 | 0.999 | 110.00 | 19.55 | 1.01 |  | 0.160 | 0.532 | 0.999 | 78.48 | 7.81 | 2.71 |
| DM-THM ^e^ | 278.1>132 | 278.1>197.1 | 3.25 | 0.302 | 1.007 | 0.999 | 98.67 | 14.82 | 0.14 |  | - | - | - | - | - | - |
| FLO ^a^ | 230>148.1 | 230>203.1 | 3.35 | - | - | - | - | - | - |  | - | - | - | - | - | - |
| THCP ^d^ | 253.02>126.1 | 253.02>99.1 | 3.35 | - | - | - | - | - | - |  | - | - | - | - | - | - |
| THM-*d_4_* | 297.1>134 | 297.1>214.2 | 2.92 |  |  |  |  |  |  |  |  |  |  |  |  |  |
| CLO-*d_3_* | 253.2>172.2 | 253.2>132.1 | 3.12 |  |  |  |  |  |  |  |  |  |  |  |  |  |
| IMI-*d_4_* | 260.2>179.2 | 260.2>213.1 | 3.17 |  |  |  |  |  |  |  |  |  |  |  |  |  |
| ACE-*d_3_* | 226.2>126 | 226.2>59.1 | 3.20 |  |  |  |  |  |  |  |  |  |  |  |  |  |
| THCP-*d_4_* | 257.1>126 | 257.1>190.1 | 3.35 |  |  |  |  |  |  |  |  |  |  |  |  |  |

**Note:** “–” indicates compounds that were excluded from quantification in this study due to analytical limitations (e.g., high background signals or poor calibration curve fitting) or were not detected in a given biological matrix. Among the 18 target analytes, 14 were successfully quantified in at least one biological matrix (urine or semen). Four compounds (FLO, THM, Descyno-ACE, THCP-amide) were excluded from quantification due to analytical limitations. R2: Linear regression without weighting was applied. The zero-point was included but the regression was not forced through the origin. a corrected by ACE-d3. b corrected by CLO-d3. c corrected by IMI-d4. d corrected by THCP-d4. e corrected by THM-d4. Abbreviations: DN, Desnitro-dinotefuran; UF, Dinotefuran-urea; DM-ACE, Desmethyl-acetamiprid; DN-IMI-olefin, Desnitro-imidacloprid-olefin; DN-IMI, Desnitro-imidacloprid; IMI-urea, Imidacloprid-urea; 5-OH-IMI, 5-Hydroxy-imidacloprid; DM-THM, Desmethyl-thiamethoxam; DNT, Dinotefuran; ACE, Acetamiprid; CLO, Clothianidin; THCP, Thiacloprid; IMI, Imidacloprid; IMIT, Imidaclothiz; RT, Retention time; LOD, Limit of Detection; LOQ, Limit of Quantification; ME, Matrix effects.

Table S3. Convergence diagnostics of Bayesian kernel machine regression (BKMR) models based on Gelman–Rubin statistics.

| **Outcomes** | **Urine** | **Semen** |
| --- | --- | --- |
| Sperm volume | 1.00-1.02 | 1.00-1.01 |
| Sperm concentration | 1.00-1.04 | 1.00-1.02 |
| Sperm count | 1.00-1.08 | 1.00-1.01 |
| Progressive motility | 1.00-1.00 | 1.00-1.01 |
| Total motility | 1.00-1.01 | 1.00-1.00 |
| Sperm morphology | 1.00-1.02 | 1.00-1.00 |

**Notes:** BKMR, Bayesian kernel machine regression. Values in the table represent the range of the Gelman–Rubin diagnostic (R-hat) for all model parameters (including beta and sigma).An R-hat value below 1.10 is widely accepted as an indicator of adequate MCMC chain convergence. All models in this study yielded R-hat values between 1.00 and 1.08, confirming stable posterior estimation. All models were fitted using 10,000 MCMC iterations.

Table S4. The associations of individual NEO concentrations in urine (n = 179) and semen (n = 173) with semen quality.

| **Exposure** |  | **Sperm volume** | | **Sperm concentration** | | **Sperm count** | | **Progressive motility** | | **Total motility** | | **Sperm morphology** | |
| --- | --- | --- | --- | --- | --- | --- | --- | --- | --- | --- | --- | --- | --- |
|  |  | **β (95% CI)** | ***P*** | **β (95% CI)** | ***P*** | **β (95% CI)** | ***P*** | **β (95% CI)** | ***P*** | **β (95% CI)** | ***P*** | **β (95% CI)** | ***P*** |
| **Urine** |  |  |  |  |  |  |  |  |  |  |  |  |  |
| **DN** | Continuous | 0.06 (-0.04, 0.17) | 0.246 | -0.07 (-0.35, 0.20) | 0.595 | -0.02 (-0.31, 0.28) | 0.913 | -0.19 (-0.35, -0.02) | 0.029 | -0.13 (-0.26, -0.01) | 0.044 | -0.01 (-0.14, 0.14) | 0.995 |
|  | Q1 | reference |  | reference |  | reference |  | reference |  | reference |  | reference |  |
|  | Q2 | -0.08 (-0.26, 0.10) | 0.383 | 0.14 (-0.32, 0.61) | 0.544 | 0.04 (-0.46, 0.55) | 0.861 | 0.05 (-0.23, 0.33) | 0.736 | -0.02 (-0.23, 0.2) | 0.876 | 0.01 (-0.22, 0.25) | 0.926 |
|  | Q3 | 0.07 (-0.11, 0.25) | 0.436 | -0.01 (-0.48, 0.47) | 0.980 | 0.03 (-0.48, 0.54) | 0.897 | -0.16 (-0.44, 0.13) | 0.285 | -0.13 (-0.35, 0.09) | 0.234 | -0.05 (-0.29, 0.19) | 0.693 |
|  | Q4 | 0.15 (-0.02, 0.33) | 0.087 | 0.02 (-0.44, 0.49) | 0.916 | 0.16 (-0.33, 0.65) | 0.524 | -0.30 (-0.58, -0.02) | 0.036 | -0.21 (-0.43, -0.0) | 0.049 | -0.02 (-0.26, 0.21) | 0.839 |
|  | *P* for trend | 0.033 |  | 0.924 |  | 0.551 |  | 0.015 |  | 0.028 |  | 0.729 |  |
| **UF** | Continuous | 0.03 (-0.03, 0.08) | 0.314 | -0.13 (-0.27, 0.01) | 0.066 | -0.1 (-0.25, 0.05) | 0.18 | -0.05 (-0.14, 0.03) | 0.221 | -0.04 (-0.11, 0.02) | 0.187 | -0.04 (-0.11, 0.03) | 0.305 |
|  | Q1 | reference |  | reference |  | reference |  | reference |  | reference |  | reference |  |
|  | Q2 | -0.09 (-0.26, 0.08) | 0.305 | -0.09 (-0.55, 0.37) | 0.689 | -0.16 (-0.64, 0.33) | 0.526 | -0.04 (-0.32, 0.24) | 0.766 | -0.06 (-0.27, 0.16) | 0.604 | -0.07 (-0.3, 0.16) | 0.555 |
|  | Q3 | -0.19 (-0.36, -0.01) | 0.034 | -0.39 (-0.86, 0.07) | 0.097 | -0.57 (-1.06, -0.07) | 0.024 | -0.22 (-0.50, 0.07) | 0.130 | -0.16 (-0.37, 0.06) | 0.151 | -0.01 (-0.25, 0.22) | 0.930 |
|  | Q4 | 0.12 (-0.06, 0.29) | 0.183 | -0.26 (-0.72, 0.2) | 0.261 | -0.14 (-0.63, 0.35) | 0.577 | -0.25 (-0.53, 0.03) | 0.081 | -0.24 (-0.45, -0.03) | 0.027 | -0.08 (-0.31, 0.16) | 0.515 |
|  | *P* for trend | 0.362 |  | 0.151 |  | 0.318 |  | 0.044 |  | 0.018 |  | 0.633 |  |
| **DN-IMI-olefin** | Continuous | 0.07 (0.01, 0.14) | 0.029 | -0.11 (-0.28, 0.06) | 0.200 | -0.04 (-0.23, 0.14) | 0.648 | -0.1 (-0.21, 0.01) | 0.051 | -0.09 (-0.17, -0.01) | 0.028 | -0.07 (-0.16, 0.01) | 0.100 |
|  | Q1 | reference |  | reference |  | reference |  | reference |  | reference |  | reference |  |
|  | Q2 | 0.19 (0.02, 0.37) | 0.029 | -0.03 (-0.49, 0.43) | 0.898 | 0.15 (-0.35, 0.64) | 0.56 | -0.0 (-0.29, 0.28) | 0.981 | -0.01 (-0.23, 0.2) | 0.913 | 0.01 (-0.22, 0.24) | 0.928 |
|  | Q3 | 0.18 (0.01, 0.35) | 0.049 | 0.11 (-0.35, 0.57) | 0.635 | 0.26 (-0.23, 0.76) | 0.293 | -0.09 (-0.37, 0.20) | 0.550 | -0.04 (-0.25, 0.18) | 0.727 | 0.09 (-0.15, 0.32) | 0.468 |
|  | Q4 | 0.21 (0.03, 0.38) | 0.024 | -0.18 (-0.65, 0.29) | 0.455 | 0.01 (-0.49, 0.51) | 0.976 | -0.15 (-0.44, 0.13) | 0.292 | -0.16 (-0.38, 0.06) | 0.153 | -0.11 (-0.35, 0.13) | 0.357 |
|  | *P* for trend | 0.036 |  | 0.612 |  | 0.843 |  | 0.240 |  | 0.155 |  | 0.515 |  |
| **DM-ACE** | Continuous | 0.02 (-0.03, 0.07) | 0.405 | -0.08 (-0.20, 0.05) | 0.231 | -0.05 (-0.19, 0.08) | 0.422 | 0.02 (-0.05, 0.10) | 0.559 | 0.02 (-0.04, 0.07) | 0.585 | -0.01 (-0.08, 0.05) | 0.676 |
|  | Q1 | reference |  | reference |  | reference |  | reference |  | reference |  | reference |  |
|  | Q2 | 0.10 (-0.08, 0.27) | 0.284 | -0.05 (-0.51, 0.41) | 0.825 | 0.03 (-0.46, 0.52) | 0.906 | 0.13 (-0.15, 0.42) | 0.345 | 0.12 (-0.09, 0.33) | 0.270 | 0.26 (0.03, 0.48) | 0.026 |
|  | Q3 | 0.03 (-0.14, 0.21) | 0.700 | 0.01 (-0.44, 0.47) | 0.949 | 0.04 (-0.45, 0.53) | 0.884 | 0.17 (-0.11, 0.45) | 0.230 | 0.12 (-0.09, 0.33) | 0.269 | 0.02 (-0.21, 0.25) | 0.865 |
|  | Q4 | 0.16 (-0.02, 0.34) | 0.074 | -0.33 (-0.79, 0.14) | 0.164 | -0.17 (-0.66, 0.33) | 0.508 | -0.04 (-0.33, 0.24) | 0.756 | -0.05 (-0.26, 0.17) | 0.664 | -0.06 (-0.29, 0.16) | 0.585 |
|  | *P* for trend | 0.139 |  | 0.224 |  | 0.543 |  | 0.859 |  | 0.711 |  | 0.273 |  |
| **DN-IMI** | Continuous | -0.01 (-0.04, 0.02) | 0.427 | -0.03 (-0.10, 0.05) | 0.49 | -0.04 (-0.11, 0.04) | 0.388 | 0.0 (-0.04, 0.05) | 0.844 | 0.02 (-0.02, 0.05) | 0.296 | 0.01 (-0.03, 0.05) | 0.525 |
|  | Q1 | reference |  | reference |  | reference |  | reference |  | reference |  | reference |  |
|  | Q2 | 0.23 (0.06, 0.40) | 0.010 | -0.07 (-0.53, 0.39) | 0.756 | 0.13 (-0.36, 0.62) | 0.599 | 0.02 (-0.26, 0.30) | 0.887 | 0.03 (-0.18, 0.25) | 0.749 | -0.14 (-0.37, 0.09) | 0.224 |
|  | Q3 | 0.10 (-0.07, 0.28) | 0.251 | 0.04 (-0.42, 0.51) | 0.860 | 0.16 (-0.33, 0.66) | 0.511 | 0.03 (-0.26, 0.32) | 0.833 | 0.07 (-0.15, 0.29) | 0.517 | 0.01 (-0.22, 0.24) | 0.926 |
|  | Q4 | 0.01 (-0.17, 0.18) | 0.972 | -0.23 (-0.70, 0.23) | 0.328 | -0.24 (-0.73, 0.26) | 0.348 | 0.01 (-0.29, 0.29) | 0.995 | 0.10 (-0.12, 0.32) | 0.383 | -0.01 (-0.24, 0.22) | 0.940 |
|  | *P* for trend | 0.695 |  | 0.448 |  | 0.413 |  | 0.973 |  | 0.344 |  | 0.727 |  |
| **IMI-urea** | Continuous | 0.02 (-0.05, 0.08) | 0.587 | -0.10 (-0.27, 0.06) | 0.222 | -0.1 (-0.27, 0.08) | 0.286 | -0.08 (-0.18, 0.02) | 0.117 | -0.04 (-0.12, 0.03) | 0.272 | -0.04 (-0.12, 0.05) | 0.402 |
|  | Q1 | reference |  | reference |  | reference |  | reference |  | reference |  | reference |  |
|  | Q2 | -0.03 (-0.21, 0.16) | 0.763 | -0.69 (-1.15, -0.22) | 0.004 | -0.73 (-1.22, -0.23) | 0.004 | 0.05 (-0.24, 0.34) | 0.722 | 0.05 (-0.16, 0.27) | 0.631 | -0.12 (-0.35, 0.12) | 0.340 |
|  | Q3 | -0.02 (-0.20, 0.16) | 0.810 | -0.35 (-0.81, 0.11) | 0.136 | -0.39 (-0.88, 0.10) | 0.116 | 0.08 (-0.21, 0.36) | 0.587 | 0.10 (-0.12, 0.32) | 0.369 | -0.07 (-0.30, 0.17) | 0.576 |
|  | Q4 | 0.01 (-0.18, 0.18) | 0.994 | -0.34 (-0.80, 0.12) | 0.142 | -0.35 (-0.84, 0.13) | 0.154 | -0.25 (-0.54, 0.03) | 0.079 | -0.16 (-0.38, 0.05) | 0.134 | -0.16 (-0.39, 0.08) | 0.186 |
|  | *P* for trend | 0.984 |  | 0.313 |  | 0.318 |  | 0.113 |  | 0.202 |  | 0.248 |  |
| **5-OH-IMI** | Continuous | -0.01 (-0.06, 0.04) | 0.694 | -0.13 (-0.25, -0.01) | 0.036 | -0.13 (-0.26, -0.01) | 0.044 | -0.01 (-0.09, 0.06) | 0.761 | 0.0 (-0.06, 0.06) | 0.926 | -0.07 (-0.13, -0.01) | 0.024 |
|  | Q1 | reference |  | reference |  | reference |  | reference |  | reference |  | reference |  |
|  | Q2 | -0.01 (-0.18, 0.17) | 0.960 | 0.07 (-0.39, 0.53) | 0.758 | 0.06 (-0.43, 0.56) | 0.801 | 0.01 (-0.28, 0.28) | 0.997 | -0.02 (-0.24, 0.19) | 0.837 | -0.12 (-0.35, 0.11) | 0.301 |
|  | Q3 | -0.01 (-0.20, 0.17) | 0.881 | 0.10 (-0.37, 0.57) | 0.665 | 0.11 (-0.39, 0.61) | 0.671 | 0.16 (-0.13, 0.44) | 0.285 | 0.15 (-0.07, 0.37) | 0.182 | 0.01 (-0.23, 0.24) | 0.955 |
|  | Q4 | -0.05 (-0.23, 0.13) | 0.580 | -0.22 (-0.68, 0.24) | 0.353 | -0.24 (-0.73, 0.26) | 0.348 | -0.03 (-0.31, 0.26) | 0.842 | 0.02 (-0.20, 0.23) | 0.874 | -0.11 (-0.34, 0.12) | 0.346 |
|  | *P* for trend | 0.248 |  | 0.405 |  | 0.407 |  | 0.901 |  | 0.553 |  | 0.554 |  |
| **ACE** | Continuous | 0.02 (-0.08, 0.12) | 0.692 | -0.08 (-0.34, 0.18) | 0.565 | -0.06 (-0.34, 0.22) | 0.664 | -0.08 (-0.24, 0.08) | 0.328 | -0.06 (-0.18, 0.06) | 0.313 | 0.03 (-0.10, 0.16) | 0.632 |
|  | Q1 | reference |  | reference |  | reference |  | reference |  | reference |  | reference |  |
|  | Q2 | 0.12 (-0.05, 0.30) | 0.166 | -0.34 (-0.8, 0.11) | 0.141 | -0.24 (-0.73, 0.25) | 0.336 | 0.02 (-0.26, 0.30) | 0.897 | -0.02 (-0.24, 0.19) | 0.831 | -0.12 (-0.35, 0.11) | 0.293 |
|  | Q3 | 0.06 (-0.12, 0.24) | 0.514 | -0.11 (-0.57, 0.35) | 0.647 | -0.08 (-0.57, 0.41) | 0.748 | 0.06 (-0.22, 0.35) | 0.665 | 0.03 (-0.19, 0.24) | 0.805 | 0.10 (-0.13, 0.33) | 0.402 |
|  | Q4 | 0.05 (-0.13, 0.23) | 0.581 | -0.21 (-0.68, 0.25) | 0.363 | -0.18 (-0.67, 0.32) | 0.478 | -0.08 (-0.37, 0.20) | 0.576 | -0.08 (-0.29, 0.14) | 0.485 | 0.02 (-0.21, 0.26) | 0.832 |
|  | *P* for trend | 0.767 |  | 0.586 |  | 0.636 |  | 0.665 |  | 0.605 |  | 0.425 |  |
| **CLO** | Continuous | 0.02 (-0.02, 0.07) | 0.321 | -0.09 (-0.22, 0.04) | 0.161 | -0.06 (-0.20, 0.08) | 0.404 | 0.02 (-0.06, 0.09) | 0.708 | 0.01 (-0.06, 0.07) | 0.868 | -0.01 (-0.08, 0.05) | 0.731 |
|  | Q1 | reference |  | reference |  | reference |  | reference |  | reference |  | reference |  |
|  | Q2 | 0.08 (-0.10, 0.26) | 0.379 | -0.08 (-0.54, 0.38) | 0.736 | 0.01 (-0.49, 0.50) | 0.987 | -0.01 (-0.30, 0.28) | 0.950 | -0.03 (-0.25, 0.19) | 0.788 | 0.07 (-0.16, 0.30) | 0.549 |
|  | Q3 | 0.17 (-0.02, 0.35) | 0.075 | -0.18 (-0.66, 0.29) | 0.452 | 0.02 (-0.49, 0.53) | 0.929 | -0.01 (-0.31, 0.28) | 0.926 | -0.01 (-0.24, 0.21) | 0.915 | -0.13 (-0.37, 0.11) | 0.289 |
|  | Q4 | 0.17 (-0.01, 0.35) | 0.060 | -0.45 (-0.92, 0.01) | 0.057 | -0.26 (-0.76, 0.25) | 0.314 | 0.04 (-0.25, 0.33) | 0.782 | 0.0 (-0.22, 0.23) | 0.966 | -0.03 (-0.27, 0.21) | 0.806 |
|  | *P* for trend | 0.038 |  | 0.050 |  | 0.341 |  | 0.795 |  | 0.923 |  | 0.453 |  |
| **IMI** | Continuous | 0.05 (-0.01, 0.11) | 0.099 | -0.03 (-0.18, 0.13) | 0.716 | 0.01 (-0.15, 0.18) | 0.894 | -0.02 (-0.11, 0.08) | 0.732 | -0.03 (-0.10, 0.04) | 0.450 | -0.05 (-0.13, 0.02) | 0.178 |
|  | Q1 | reference |  | reference |  | reference |  | reference |  | reference |  | reference |  |
|  | Q2 | 0.20 (0.02, 0.37) | 0.026 | 0.05 (-0.41, 0.50) | 0.843 | 0.23 (-0.26, 0.72) | 0.359 | -0.15 (-0.43, 0.13) | 0.295 | -0.13 (-0.34, 0.08) | 0.228 | -0.13 (-0.36, 0.10) | 0.273 |
|  | Q3 | 0.16 (-0.02, 0.34) | 0.083 | -0.15 (-0.61, 0.32) | 0.535 | -0.01 (-0.50, 0.49) | 0.990 | -0.15 (-0.44, 0.13) | 0.295 | -0.11 (-0.32, 0.11) | 0.329 | 0.02 (-0.22, 0.25) | 0.895 |
|  | Q4 | 0.12 (-0.06, 0.30) | 0.178 | -0.18 (-0.64, 0.28) | 0.445 | -0.09 (-0.58, 0.40) | 0.712 | -0.12 (-0.40, 0.17) | 0.418 | -0.14 (-0.35, 0.08) | 0.205 | -0.17 (-0.4, 0.06) | 0.138 |
|  | *P* for trend | 0.268 |  | 0.327 |  | 0.522 |  | 0.444 |  | 0.256 |  | 0.296 |  |
| **IMIT** | Continuous | 0.02 (-0.05, 0.10) | 0.521 | 0.12 (-0.07, 0.31) | 0.225 | 0.12 (-0.08, 0.33) | 0.234 | -0.01 (-0.13, 0.11) | 0.848 | 0.02 (-0.07, 0.11) | 0.706 | 0.03 (-0.07, 0.13) | 0.536 |
|  | Q1 | reference |  | reference |  | reference |  | reference |  | reference |  | reference |  |
|  | Q2 | 0.13 (-0.06, 0.31) | 0.175 | 0.29 (-0.18, 0.76) | 0.228 | 0.40 (-0.10, 0.90) | 0.118 | 0.17 (-0.12, 0.46) | 0.254 | 0.13 (-0.09, 0.35) | 0.250 | -0.07 (-0.31, 0.17) | 0.545 |
|  | Q3 | 0.02 (-0.16, 0.20) | 0.852 | -0.13 (-0.59, 0.33) | 0.579 | -0.13 (-0.62, 0.36) | 0.601 | 0.19 (-0.10, 0.47) | 0.195 | 0.15 (-0.07, 0.37) | 0.178 | -0.16 (-0.39, 0.08) | 0.184 |
|  | Q4 | 0.07 (-0.12, 0.25) | 0.467 | 0.25 (-0.23, 0.72) | 0.305 | 0.29 (-0.21, 0.79) | 0.254 | -0.06 (-0.35, 0.24) | 0.707 | 0.01 (-0.21, 0.23) | 0.910 | -0.03 (-0.27, 0.21) | 0.827 |
|  | *P* for trend | 0.759 |  | 0.694 |  | 0.695 |  | 0.767 |  | 0.860 |  | 0.653 |  |
| **Semen** |  |  |  |  |  |  |  |  |  |  |  |  |  |
| **DN** | Continuous | 0.22 (0.07, 0.38) | 0.005 | 0.06 (-0.37, 0.48) | 0.793 | 0.28 (-0.17, 0.73) | 0.227 | -0.1 (-0.35, 0.15) | 0.433 | -0.07 (-0.25, 0.11) | 0.464 | 0.04 (-0.17, 0.25) | 0.713 |
|  | Q1 | reference |  | reference |  | reference |  | reference |  | reference |  | reference |  |
|  | Q2 | 0.01 (-0.17, 0.18) | 0.996 | 0.36 (-0.1, 0.83) | 0.128 | 0.35 (-0.15, 0.85) | 0.170 | 0.18 (-0.10, 0.46) | 0.209 | 0.09 (-0.11, 0.29) | 0.377 | 0.01 (-0.23, 0.24) | 0.942 |
|  | Q3 | 0.07 (-0.11, 0.25) | 0.457 | 0.16 (-0.32, 0.63) | 0.521 | 0.22 (-0.30, 0.73) | 0.401 | -0.01 (-0.30, 0.27) | 0.923 | 0.02 (-0.19, 0.22) | 0.874 | 0.06 (-0.18, 0.30) | 0.607 |
|  | Q4 | 0.14 (-0.05, 0.32) | 0.141 | 0.08 (-0.41, 0.56) | 0.757 | 0.21 (-0.32, 0.73) | 0.436 | -0.08 (-0.38, 0.21) | 0.573 | -0.06 (-0.27, 0.15) | 0.581 | 0.03 (-0.21, 0.28) | 0.803 |
|  | *P* for trend | 0.106 |  | 0.967 |  | 0.557 |  | 0.356 |  | 0.464 |  | 0.712 |  |
| **DN-IMI** | Continuous | 0.04 (-0.02, 0.09) | 0.175 | -0.17 (-0.31, -0.03) | 0.018 | -0.14 (-0.29, 0.01) | 0.075 | -0.01 (-0.09, 0.08) | 0.828 | -0.01 (-0.07, 0.06) | 0.859 | -0.03 (-0.10, 0.04) | 0.392 |
|  | Q1 | reference |  | reference |  | reference |  | reference |  | reference |  | reference |  |
|  | Q2 | 0.10 (-0.08, 0.27) | 0.266 | -0.03 (-0.48, 0.43) | 0.911 | 0.08 (-0.42, 0.57) | 0.763 | -0.05 (-0.32, 0.23) | 0.746 | -0.02 (-0.22, 0.18) | 0.862 | -0.06 (-0.30, 0.17) | 0.588 |
|  | Q3 | 0.12 (-0.05, 0.30) | 0.161 | -0.06 (-0.52, 0.39) | 0.781 | 0.04 (-0.45, 0.53) | 0.864 | 0.17 (-0.11, 0.45) | 0.225 | 0.12 (-0.08, 0.32) | 0.234 | 0.01 (-0.23, 0.23) | 0.977 |
|  | Q4 | 0.06 (-0.12, 0.23) | 0.516 | -0.56 (-1.02, -0.10) | 0.017 | -0.51 (-1.00, -0.02) | 0.041 | -0.08 (-0.36, 0.20) | 0.573 | -0.06 (-0.26, 0.14) | 0.570 | -0.11 (-0.35, 0.12) | 0.342 |
|  | *P* for trend | 0.471 |  | 0.022 |  | 0.053 |  | 0.938 |  | 0.898 |  | 0.461 |  |
| **IMI-urea** | Continuous | -0.01 (-0.08, 0.06) | 0.746 | -0.21 (-0.40, -0.02) | 0.029 | -0.22 (-0.43, -0.02) | 0.033 | -0.07 (-0.19, 0.04) | 0.203 | -0.09 (-0.17, -0.01) | 0.037 | -0.09 (-0.18, 0.01) | 0.078 |
|  | Q1 | reference |  | reference |  | reference |  | reference |  | reference |  | reference |  |
|  | Q2 | 0.01 (-0.17, 0.19) | 0.915 | -0.57 (-1.04, -0.11) | 0.016 | -0.57 (-1.07, -0.07) | 0.026 | -0.07 (-0.35, 0.21) | 0.626 | -0.16 (-0.36, 0.04) | 0.112 | -0.15 (-0.39, 0.08) | 0.206 |
|  | Q3 | -0.05 (-0.22, 0.13) | 0.610 | -0.37 (-0.83, 0.10) | 0.120 | -0.40 (-0.90, 0.10) | 0.112 | 0.04 (-0.24, 0.32) | 0.793 | -0.06 (-0.26, 0.14) | 0.528 | -0.05 (-0.29, 0.18) | 0.645 |
|  | Q4 | -0.01 (-0.20, 0.19) | 0.957 | -0.48 (-0.98, 0.03) | 0.063 | -0.47 (-1.01, 0.07) | 0.087 | -0.24 (-0.55, 0.06) | 0.115 | -0.26 (-0.48, -0.04) | 0.019 | -0.19 (-0.44, 0.07) | 0.145 |
|  | *P* for trend | 0.768 |  | 0.103 |  | 0.116 |  | 0.265 |  | 0.060 |  | 0.260 |  |
| **ACE** | Continuous | 0.06 (0.02, 0.1) | 0.004 | -0.05 (-0.15, 0.06) | 0.41 | 0.01 (-0.11, 0.12) | 0.901 | -0.02 (-0.08, 0.05) | 0.628 | -0.03 (-0.08, 0.02) | 0.187 | 0.01 (-0.05, 0.06) | 0.844 |
|  | Q1 | reference |  | reference |  | reference |  | reference |  | reference |  | reference |  |
|  | Q2 | 0.10 (-0.07, 0.26) | 0.237 | 0.11 (-0.34, 0.57) | 0.624 | 0.19 (-0.31, 0.69) | 0.455 | 0.06 (-0.22, 0.34) | 0.677 | -0.02 (-0.22, 0.17) | 0.813 | -0.04 (-0.27, 0.19) | 0.761 |
|  | Q3 | 0.37 (0.21, 0.53) | <0.001 | -0.27 (-0.72, 0.19) | 0.247 | 0.08 (-0.41, 0.57) | 0.746 | -0.08 (-0.35, 0.20) | 0.582 | -0.11 (-0.30, 0.09) | 0.285 | 0.10 (-0.13, 0.33) | 0.377 |
|  | Q4 | 0.22 (0.06, 0.39) | 0.009 | -0.21 (-0.67, 0.25) | 0.368 | -0.02 (-0.52, 0.48) | 0.948 | -0.01 (-0.28, 0.28) | 0.982 | -0.08 (-0.28, 0.12) | 0.422 | 0.10 (-0.13, 0.34) | 0.383 |
|  | *P* for trend | 0.001 |  | 0.175 |  | 0.868 |  | 0.746 |  | 0.301 |  | 0.234 |  |

**Note:** Natural log-transformed exposure variables were divided into quartiles (Q1–Q4), with the lowest quartile (Q1) serving as the reference group. Models were adjusted for potential confounders, including age, body mass index (BMI), education level, smoking status, season of sample collection, abstinence duration, and occupational exposure likelihood. Effect estimates are presented as regression coefficients with 95% confidence interval. P for trend was calculated by assigning the median value of each quartile to a continuous variable and modeling it as a linear term.

Table S5. Associations of urine (n = 179) and semen (n = 173) NEO concentrations with semen quality parameters stratified by age.

| **Outcome** | **Exposure** | **Age < 35 years** | | **Age ≥ 35 years** | | ***P* for**  **interaction** |
| --- | --- | --- | --- | --- | --- | --- |
|  |  | **β (95% CI)** | ***P*** | **β (95% CI)** | ***P*** |  |
| **Urine** |  |  |  |  |  |  |
| Sperm volume | DN | 0.11 (-0.04, 0.25) | 0.160 | 0.02 (-0.15, 0.19) | 0.814 | 0.298 |
|  | UF | 0.08 (0.01, 0.16) | 0.047 | -0.01 (-0.09, 0.07) | 0.849 | 0.166 |
|  | DN-IMI-olefin | 0.08 (-0.02, 0.17) | 0.117 | 0.08 (-0.02, 0.17) | 0.115 | 0.945 |
|  | DM-ACE | -0.01 (-0.07, 0.07) | 0.957 | 0.05 (-0.03, 0.13) | 0.198 | 0.192 |
|  | DN-IMI | -0.01 (-0.05, 0.04) | 0.810 | -0.01 (-0.06, 0.04) | 0.657 | 0.527 |
|  | IMI-urea | 0.07 (-0.03, 0.16) | 0.155 | -0.02 (-0.12, 0.08) | 0.710 | 0.206 |
|  | 5-OH-IMI | -0.02 (-0.10, 0.06) | 0.583 | 0.01 (-0.06, 0.07) | 0.799 | 0.595 |
|  | ACE | 0.09 (-0.05, 0.24) | 0.196 | -0.04 (-0.19, 0.11) | 0.622 | 0.363 |
|  | CLO | 0.07 (-0.01, 0.14) | 0.084 | -0.01 (-0.07, 0.07) | 0.979 | 0.075 |
|  | IMI | 0.05 (-0.03, 0.13) | 0.228 | 0.04 (-0.06, 0.13) | 0.434 | 0.853 |
|  | IMIT | 0.04 (-0.06, 0.14) | 0.461 | -0.02 (-0.14, 0.11) | 0.776 | 0.315 |
| Sperm concentration | DN | -0.41 (-0.78, -0.05) | 0.025 | 0.31 (-0.15, 0.77) | 0.178 | 0.006 |
|  | UF | -0.08 (-0.28, 0.12) | 0.429 | -0.21 (-0.43, 0.0) | 0.055 | 0.420 |
|  | DN-IMI-olefin | -0.03 (-0.27, 0.20) | 0.775 | -0.22 (-0.49, 0.04) | 0.099 | 0.332 |
|  | DM-ACE | -0.19 (-0.35, -0.02) | 0.026 | 0.06 (-0.16, 0.27) | 0.592 | 0.090 |
|  | DN-IMI | -0.01 (-0.11, 0.09) | 0.831 | -0.06 (-0.19, 0.07) | 0.341 | 0.736 |
|  | IMI-urea | -0.1 (-0.33, 0.12) | 0.377 | -0.16 (-0.45, 0.13) | 0.270 | 0.840 |
|  | 5-OH-IMI | -0.11 (-0.30, 0.09) | 0.291 | -0.18 (-0.36, -0.01) | 0.036 | 0.519 |
|  | ACE | -0.18 (-0.53, 0.18) | 0.321 | -0.01 (-0.43, 0.42) | 0.972 | 0.638 |
|  | CLO | -0.08 (-0.27, 0.12) | 0.433 | -0.15 (-0.34, 0.04) | 0.130 | 0.827 |
|  | IMI | 0.09 (-0.11, 0.29) | 0.388 | -0.24 (-0.50, 0.02) | 0.071 | 0.058 |
|  | IMIT | 0.11 (-0.14, 0.36) | 0.382 | 0.18 (-0.16, 0.52) | 0.299 | 0.602 |
| Sperm count | DN | -0.33 (-0.71, 0.05) | 0.086 | 0.34 (-0.16, 0.84) | 0.178 | 0.021 |
|  | UF | -0.01 (-0.21, 0.21) | 0.966 | -0.21 (-0.44, 0.03) | 0.085 | 0.249 |
|  | DN-IMI-olefin | 0.02 (-0.23, 0.27) | 0.862 | -0.15 (-0.44, 0.15) | 0.319 | 0.388 |
|  | DM-ACE | -0.19 (-0.36, -0.02) | 0.027 | 0.12 (-0.12, 0.35) | 0.327 | 0.031 |
|  | DN-IMI | -0.01 (-0.12, 0.09) | 0.820 | -0.07 (-0.21, 0.07) | 0.331 | 0.586 |
|  | IMI-urea | -0.06 (-0.29, 0.18) | 0.634 | -0.18 (-0.50, 0.13) | 0.256 | 0.580 |
|  | 5-OH-IMI | -0.13 (-0.33, 0.08) | 0.231 | -0.17 (-0.36, 0.02) | 0.079 | 0.685 |
|  | ACE | -0.10 (-0.48, 0.27) | 0.578 | -0.05 (-0.51, 0.42) | 0.840 | 0.882 |
|  | CLO | -0.01 (-0.21, 0.19) | 0.910 | -0.14 (-0.35, 0.07) | 0.191 | 0.434 |
|  | IMI | 0.12 (-0.09, 0.33) | 0.248 | -0.21 (-0.49, 0.08) | 0.159 | 0.076 |
|  | IMIT | 0.13 (-0.13, 0.38) | 0.323 | 0.14 (-0.24, 0.51) | 0.471 | 0.924 |
| Progressive motility | DN | -0.17 (-0.40, 0.06) | 0.155 | -0.22 (-0.49, 0.04) | 0.097 | 0.897 |
|  | UF | -0.04 (-0.16, 0.09) | 0.580 | -0.07 (-0.19, 0.06) | 0.302 | 0.933 |
|  | DN-IMI-olefin | -0.03 (-0.19, 0.12) | 0.652 | -0.18 (-0.33, -0.03) | 0.021 | 0.273 |
|  | DM-ACE | 0.04 (-0.06, 0.15) | 0.421 | -0.02 (-0.14, 0.11) | 0.763 | 0.532 |
|  | DN-IMI | 0.03 (-0.03, 0.09) | 0.342 | -0.01 (-0.08, 0.07) | 0.823 | 0.424 |
|  | IMI-urea | 0.01 (-0.13, 0.16) | 0.838 | -0.22 (-0.38, -0.06) | 0.008 | 0.043 |
|  | 5-OH-IMI | 0.04 (-0.09, 0.17) | 0.534 | -0.06 (-0.16, 0.04) | 0.236 | 0.185 |
|  | ACE | 0.05 (-0.17, 0.28) | 0.65 | -0.26 (-0.5, -0.02) | 0.032 | 0.073 |
|  | CLO | 0.01 (-0.12, 0.13) | 0.918 | -0.01 (-0.12, 0.11) | 0.947 | 0.935 |
|  | IMI | 0.02 (-0.11, 0.15) | 0.785 | -0.13 (-0.28, 0.03) | 0.102 | 0.257 |
|  | IMIT | 0.05 (-0.11, 0.2)0 | 0.559 | -0.10 (-0.30, 0.10) | 0.312 | 0.255 |
| Total motility | DN | -0.10 (-0.29, 0.09) | 0.306 | -0.16 (-0.34, 0.03) | 0.091 | 0.941 |
|  | UF | -0.04 (-0.14, 0.06) | 0.443 | -0.04 (-0.13, 0.05) | 0.38 | 0.812 |
|  | DN-IMI-olefin | -0.06 (-0.18, 0.06) | 0.336 | -0.11 (-0.22, -0.01) | 0.038 | 0.709 |
|  | DM-ACE | 0.03 (-0.05, 0.12) | 0.431 | -0.02 (-0.11, 0.07) | 0.675 | 0.442 |
|  | DN-IMI | 0.04 (-0.01, 0.09) | 0.143 | 0.01 (-0.04, 0.06) | 0.717 | 0.414 |
|  | IMI-urea | 0.01 (-0.11, 0.12) | 0.908 | -0.11 (-0.23, 0.01) | 0.053 | 0.183 |
|  | 5-OH-IMI | 0.02 (-0.08, 0.13) | 0.626 | -0.03 (-0.10, 0.04) | 0.398 | 0.344 |
|  | ACE | 0.03 (-0.15, 0.21) | 0.741 | -0.18 (-0.35, -0.01) | 0.035 | 0.139 |
|  | CLO | 0.01 (-0.09, 0.10) | 0.919 | -0.01 (-0.09, 0.07) | 0.798 | 0.868 |
|  | IMI | -0.01 (-0.11, 0.10) | 0.875 | -0.11 (-0.21, -0.01) | 0.044 | 0.308 |
|  | IMIT | 0.06 (-0.07, 0.19) | 0.344 | -0.06 (-0.20, 0.08) | 0.380 | 0.235 |
| Sperm morphology | DN | -0.06 (-0.23, 0.12) | 0.525 | 0.09 (-0.14, 0.32) | 0.428 | 0.155 |
|  | UF | -0.07 (-0.16, 0.02) | 0.136 | 0.01 (-0.10, 0.12) | 0.821 | 0.173 |
|  | DN-IMI-olefin | -0.10 (-0.21, 0.01) | 0.065 | -0.04 (-0.18, 0.09) | 0.509 | 0.606 |
|  | DM-ACE | -0.05 (-0.13, 0.03) | 0.232 | 0.04 (-0.06, 0.15) | 0.421 | 0.206 |
|  | DN-IMI | 0.02 (-0.02, 0.07) | 0.300 | 0.02 (-0.04, 0.08) | 0.536 | 0.980 |
|  | IMI-urea | -0.07 (-0.17, 0.04) | 0.196 | -0.03 (-0.17, 0.11) | 0.672 | 0.719 |
|  | 5-OH-IMI | -0.07 (-0.16, 0.02) | 0.123 | -0.10 (-0.19, -0.02) | 0.017 | 0.662 |
|  | ACE | -0.11 (-0.28, 0.05) | 0.172 | 0.19 (-0.01, 0.40) | 0.061 | 0.003 |
|  | CLO | -0.02 (-0.11, 0.07) | 0.725 | 0.01 (-0.09, 0.11) | 0.828 | 0.805 |
|  | IMI | -0.08 (-0.18, 0.01) | 0.076 | -0.05 (-0.18, 0.08) | 0.426 | 0.440 |
|  | IMIT | 0.03 (-0.08, 0.15) | 0.596 | 0.03 (-0.14, 0.20) | 0.749 | 0.839 |
| **Semen** |  |  |  |  |  |  |
| Sperm volume | DN | 0.31 (0.09, 0.53) | 0.007 | 0.11 (-0.14, 0.35) | 0.386 | 0.857 |
|  | DN-IMI | 0.07 (-0.01, 0.14) | 0.069 | 0.01 (-0.06, 0.09) | 0.752 | 0.403 |
|  | IMI-urea | 0.05 (-0.06, 0.16) | 0.380 | -0.07 (-0.17, 0.04) | 0.211 | 0.976 |
|  | ACE | 0.05 (-0.01, 0.1) | 0.086 | 0.08 (0.01, 0.14) | 0.023 | 0.335 |
| Sperm concentration | DN | 0.06 (-0.57, 0.68) | 0.861 | 0.22 (-0.46, 0.89) | 0.522 | 0.812 |
|  | DN-IMI | -0.08 (-0.28, 0.13) | 0.461 | -0.26 (-0.46, -0.05) | 0.014 | 0.274 |
|  | IMI-urea | -0.20 (-0.50, 0.10) | 0.181 | -0.24 (-0.53, 0.04) | 0.094 | 0.723 |
|  | ACE | -0.12 (-0.27, 0.03) | 0.105 | 0.07 (-0.11, 0.26) | 0.433 | 0.192 |
| Sperm count | DN | 0.36 (-0.29, 1.01) | 0.276 | 0.33 (-0.42, 1.08) | 0.385 | 0.800 |
|  | DN-IMI | -0.01 (-0.22, 0.21) | 0.945 | -0.25 (-0.48, -0.02) | 0.033 | 0.187 |
|  | IMI-urea | -0.15 (-0.47, 0.16) | 0.329 | -0.31 (-0.63, 0.01) | 0.054 | 0.776 |
|  | ACE | -0.07 (-0.23, 0.08) | 0.357 | 0.14 (-0.07, 0.34) | 0.193 | 0.158 |
| Progressive motility | DN | 0.05 (-0.34, 0.43) | 0.815 | -0.16 (-0.55, 0.22) | 0.402 | 0.313 |
|  | DN-IMI | 0.05 (-0.08, 0.17) | 0.479 | -0.05 (-0.17, 0.07) | 0.367 | 0.360 |
|  | IMI-urea | -0.09 (-0.28, 0.09) | 0.323 | -0.08 (-0.25, 0.08) | 0.332 | 0.662 |
|  | ACE | -0.02 (-0.11, 0.07) | 0.675 | -0.01 (-0.11, 0.10) | 0.922 | 0.689 |
| Total motility | DN | -0.03 (-0.31, 0.26) | 0.856 | -0.01 (-0.28, 0.25) | 0.920 | 0.612 |
|  | DN-IMI | 0.01 (-0.08, 0.11) | 0.767 | -0.02 (-0.10, 0.06) | 0.590 | 0.692 |
|  | IMI-urea | -0.13 (-0.26, 0.0) | 0.059 | -0.07 (-0.18, 0.04) | 0.231 | 0.410 |
|  | ACE | -0.05 (-0.12, 0.01) | 0.109 | 0.01 (-0.07, 0.07) | 0.990 | 0.755 |
| Sperm morphology | DN | 0.10 (-0.20, 0.39) | 0.507 | -0.02 (-0.33, 0.30) | 0.922 | 0.678 |
|  | DN-IMI | -0.03 (-0.13, 0.06) | 0.481 | -0.03 (-0.13, 0.06) | 0.490 | 0.861 |
|  | IMI-urea | -0.14 (-0.28, -0.01) | 0.044 | -0.01 (-0.14, 0.13) | 0.994 | 0.185 |
|  | ACE | -0.04 (-0.11, 0.03) | 0.310 | 0.06 (-0.03, 0.14) | 0.174 | 0.174 |

**Note:** Models were adjusted for potential confounders, including age, body mass index (BMI), education level, smoking status, season of sample collection, abstinence duration, and occupational exposure likelihood.

Table S6. Posterior inclusion probabilities (PIPs) for the associations between urinary and seminal NEO concentrations and semen quality parameters in BKMR models.

| **Outcomes** | **Sperm volume** | **Sperm concentration** | **Sperm count** | **Progressive motility** | **Total motility** | **Sperm morphology** |
| --- | --- | --- | --- | --- | --- | --- |
| **Urine** |  |  |  |  |  |  |
| DN | 0.032 | 0.050 | 0.992 | 0.178 | 0.076 | 0.091 |
| UF | 0.050 | 0.116 | 0.869 | 0.025 | 0.045 | 0.103 |
| DN-IMI-olefin | 0.190 | 0.091 | 0.923 | 0.071 | 0.257 | 0.047 |
| DM-ACE | 0.008 | 0.058 | 0.837 | 0.011 | 0.041 | 0.098 |
| DN-IMI | 0.043 | 0.059 | 0.888 | 0.017 | 0.085 | 0.126 |
| IMI-urea | 0.030 | 0.068 | 0.956 | 0.169 | 0.037 | 0.155 |
| 5-OH-IMI | 0.016 | 0.162 | 0.855 | 0 | 0 | 0.789 |
| ACE | 0.001 | 0.037 | 0.868 | 0.010 | 0.046 | 0.032 |
| CLO | 0.042 | 0.036 | 1 | 0.015 | 0.002 | 0.053 |
| IMI | 0.099 | 0.134 | 0.904 | 0.012 | 0.012 | 0.037 |
| IMIT | 0.006 | 0.049 | 0.940 | 0.014 | 0.114 | 0.034 |
| **Semen** |  |  |  |  |  |  |
| DN | 0.441 | 0.729 | 0.510 | 0.045 | 0.015 | 0.157 |
| DN-IMI | 0.102 | 0.913 | 0.612 | 0.038 | 0.017 | 0.223 |
| IMI-urea | 0.107 | 0.679 | 0.497 | 0.065 | 0.130 | 0.171 |
| ACE | 0.702 | 0.665 | 0.254 | 0.065 | 0.052 | 0.112 |

**Notes:** BKMR, Bayesian kernel machine regression; NEOs, neonicotinoids; PIP, posterior inclusion probability. PIP values range from 0 to 1, with higher values (typically > 0.5) indicating a greater relative contribution of the specific compound to the overall mixture effect.

Table S7. Associations of NEO concentrations in semen and urine with semen quality parameters in paired samples (n = 146).

| **Exposure** |  | **Sperm volume** | | **Sperm concentration** | | **Sperm count** | | **Progressive motility** | | **Total motility** | | **Sperm morphology** | |
| --- | --- | --- | --- | --- | --- | --- | --- | --- | --- | --- | --- | --- | --- |
|  |  | **β (95% CI)** | ***P*** | **β (95% CI)** | ***P*** | **β (95% CI)** | ***P*** | **β (95% CI)** | ***P*** | **β (95% CI)** | ***P*** | **β (95% CI)** | ***P*** |
| **Urine** |  |  |  |  |  |  |  |  |  |  |  |  |  |
| **DN** | Continuous | 0.07 (-0.05, 0.18) | 0.235 | -0.11 (-0.42, 0.20) | 0.500 | -0.04 (-0.37, 0.29) | 0.821 | -0.13 (-0.31, 0.05) | 0.148 | -0.08 (-0.2, 0.05) | 0.211 | 0.01 (-0.15, 0.16) | 0.938 |
|  | Q1 | reference |  | reference |  | reference |  | reference |  | reference |  | reference |  |
|  | Q2 | -0.11 (-0.30, 0.09) | 0.277 | 0.07 (-0.48, 0.61) | 0.814 | -0.05 (-0.63, 0.54) | 0.878 | 0.15 (-0.16, 0.45) | 0.346 | 0.03 (-0.19, 0.24) | 0.814 | -0.09 (-0.36, 0.18) | 0.525 |
|  | Q3 | 0.11 (-0.08, 0.31) | 0.262 | -0.03 (-0.58, 0.52) | 0.919 | 0.07 (-0.51, 0.66) | 0.804 | 0.01 (-0.30, 0.31) | 0.973 | -0.03 (-0.25, 0.19) | 0.802 | -0.06 (-0.34, 0.21) | 0.664 |
|  | Q4 | 0.15 (-0.03, 0.34) | 0.107 | 0.03 (-0.49, 0.56) | 0.902 | 0.19 (-0.38, 0.75) | 0.512 | -0.24 (-0.53, 0.06) | 0.116 | -0.15 (-0.36, 0.06) | 0.162 | -0.06 (-0.32, 0.20) | 0.648 |
|  | *P* for trend | 0.029 |  | 0.987 |  | 0.452 |  | 0.077 |  | 0.137 |  | 0.708 |  |
| **UF** | Continuous | 0.01 (-0.05, 0.07) | 0.748 | -0.14 (-0.29, 0.02) | 0.082 | -0.13 (-0.29, 0.04) | 0.136 | -0.07 (-0.16, 0.02) | 0.141 | -0.04 (-0.11, 0.02) | 0.164 | -0.04 (-0.12, 0.04) | 0.285 |
|  | Q1 | reference |  | reference |  | reference |  | reference |  | reference |  | reference |  |
|  | Q2 | -0.14 (-0.33, 0.04) | 0.13 | -0.13 (-0.65, 0.39) | 0.628 | -0.26 (-0.81, 0.29) | 0.351 | 0.02 (-0.28, 0.31) | 0.902 | -0.01 (-0.21, 0.21) | 0.992 | -0.03 (-0.29, 0.23) | 0.819 |
|  | Q3 | -0.23 (-0.42, -0.04) | 0.02 | -0.52 (-1.06, 0.01) | 0.055 | -0.75 (-1.31, -0.18) | 0.010 | -0.34 (-0.65, -0.04) | 0.026 | -0.23 (-0.45, -0.02) | 0.031 | -0.07 (-0.35, 0.20) | 0.588 |
|  | Q4 | 0.03 (-0.16, 0.22) | 0.755 | -0.27 (-0.79, 0.25) | 0.306 | -0.23 (-0.78, 0.32) | 0.408 | -0.22 (-0.51, 0.08) | 0.146 | -0.18 (-0.39, 0.02) | 0.081 | -0.06 (-0.32, 0.2) | 0.655 |
|  | *P* for trend | 0.912 |  | 0.178 |  | 0.233 |  | 0.047 |  | 0.026 |  | 0.609 |  |
| **DN-IMI-olefin** | Continuous | 0.06 (-0.01, 0.13) | 0.084 | -0.08 (-0.28, 0.11) | 0.407 | -0.02 (-0.23, 0.19) | 0.857 | -0.04 (-0.15, 0.08) | 0.517 | -0.03 (-0.1, 0.05) | 0.510 | -0.05 (-0.15, 0.04) | 0.273 |
|  | Q1 | reference |  | reference |  | reference |  | reference |  | reference |  | reference |  |
|  | Q2 | 0.17 (-0.02, 0.36) | 0.077 | 0.32 (-0.20, 0.84) | 0.229 | 0.50 (-0.06, 1.05) | 0.080 | 0.17 (-0.13, 0.47) | 0.268 | 0.13 (-0.08, 0.34) | 0.226 | 0.21 (-0.04, 0.47) | 0.102 |
|  | Q3 | 0.18 (-0.01, 0.37) | 0.066 | 0.33 (-0.19, 0.85) | 0.206 | 0.50 (-0.05, 1.05) | 0.075 | 0.02 (-0.28, 0.31) | 0.920 | 0.04 (-0.17, 0.25) | 0.694 | 0.22 (-0.03, 0.48) | 0.089 |
|  | Q4 | 0.18 (-0.01, 0.37) | 0.069 | 0.05 (-0.48, 0.58) | 0.862 | 0.23 (-0.33, 0.80) | 0.419 | -0.05 (-0.35, 0.26) | 0.767 | -0.05 (-0.27, 0.16) | 0.619 | -0.04 (-0.30, 0.22) | 0.754 |
|  | *P* for trend | 0.078 |  | 0.835 |  | 0.423 |  | 0.561 |  | 0.482 |  | 0.818 |  |
| **DM-ACE** | Continuous | 0.01 (-0.04, 0.06) | 0.612 | -0.07 (-0.21, 0.07) | 0.315 | -0.05 (-0.21, 0.10) | 0.473 | -0.01 (-0.08, 0.08) | 0.992 | -0.0 (-0.06, 0.05) | 0.868 | -0.01 (-0.08, 0.06) | 0.683 |
|  | Q1 | reference |  | reference |  | reference |  | reference |  | reference |  | reference |  |
|  | Q2 | 0.06 (-0.14, 0.25) | 0.574 | -0.04 (-0.57, 0.49) | 0.879 | 0.02 (-0.55, 0.60) | 0.935 | -0.04 (-0.34, 0.27) | 0.814 | -0.03 (-0.24, 0.19) | 0.809 | 0.22 (-0.05, 0.48) | 0.106 |
|  | Q3 | 0.03 (-0.17, 0.22) | 0.793 | -0.10 (-0.62, 0.42) | 0.706 | -0.06 (-0.62, 0.50) | 0.824 | 0.16 (-0.14, 0.45) | 0.294 | 0.10 (-0.11, 0.31) | 0.342 | 0.05 (-0.21, 0.31) | 0.713 |
|  | Q4 | 0.12 (-0.08, 0.31) | 0.229 | -0.29 (-0.81, 0.24) | 0.282 | -0.15 (-0.72, 0.41) | 0.595 | -0.13 (-0.43, 0.17) | 0.398 | -0.11 (-0.32, 0.10) | 0.301 | -0.08 (-0.34, 0.18) | 0.546 |
|  | *P* for trend | 0.295 |  | 0.279 |  | 0.554 |  | 0.705 |  | 0.559 |  | 0.374 |  |
| **DN-IMI** | Continuous | 0.0 (-0.03, 0.03) | 0.838 | -0.04 (-0.13, 0.04) | 0.308 | -0.04 (-0.13, 0.05) | 0.374 | -0.01 (-0.06, 0.04) | 0.639 | 0.01 (-0.03, 0.04) | 0.769 | -0.01 (-0.04, 0.04) | 0.978 |
|  | Q1 | reference |  | reference |  | reference |  | reference |  | reference |  | reference |  |
|  | Q2 | 0.27 (0.08, 0.46) | 0.006 | 0.06 (-0.47, 0.59) | 0.821 | 0.30 (-0.26, 0.86) | 0.293 | -0.02 (-0.33, 0.28) | 0.895 | -0.01 (-0.22, 0.21) | 0.966 | -0.15 (-0.42, 0.11) | 0.253 |
|  | Q3 | 0.17 (-0.02, 0.36) | 0.078 | 0.18 (-0.35, 0.70) | 0.505 | 0.34 (-0.22, 0.89) | 0.235 | 0.04 (-0.27, 0.34) | 0.802 | 0.04 (-0.17, 0.26) | 0.699 | -0.09 (-0.36, 0.17) | 0.500 |
|  | Q4 | 0.07 (-0.12, 0.26) | 0.459 | -0.34 (-0.86, 0.19) | 0.205 | -0.28 (-0.83, 0.28) | 0.323 | -0.11 (-0.42, 0.19) | 0.455 | -0.01 (-0.22, 0.21) | 0.982 | -0.08 (-0.35, 0.18) | 0.539 |
|  | *P* for trend | 0.727 |  | 0.290 |  | 0.374 |  | 0.558 |  | 0.901 |  | 0.671 |  |
| **IMI-urea** | Continuous | 0.01 (-0.06, 0.08) | 0.786 | -0.08 (-0.27, 0.11) | 0.381 | -0.07 (-0.27, 0.13) | 0.488 | -0.04 (-0.14, 0.07) | 0.511 | -0.01 (-0.08, 0.07) | 0.959 | -0.02 (-0.11, 0.07) | 0.674 |
|  | Q1 | reference |  | reference |  | reference |  | reference |  | reference |  | reference |  |
|  | Q2 | -0.10 (-0.30, 0.10) | 0.312 | -0.75 (-1.27, -0.23) | 0.005 | -0.84 (-1.39, -0.28) | 0.003 | 0.05 (-0.26, 0.35) | 0.761 | 0.03 (-0.18, 0.25) | 0.762 | -0.13 (-0.39, 0.14) | 0.345 |
|  | Q3 | -0.05 (-0.25, 0.15) | 0.614 | -0.36 (-0.89, 0.17) | 0.179 | -0.41 (-0.97, 0.16) | 0.155 | 0.09 (-0.21, 0.40) | 0.546 | 0.12 (-0.09, 0.34) | 0.267 | -0.11 (-0.38, 0.16) | 0.440 |
|  | Q4 | -0.03 (-0.23, 0.16) | 0.726 | -0.24 (-0.77, 0.28) | 0.357 | -0.27 (-0.82, 0.29) | 0.348 | -0.09 (-0.39, 0.22) | 0.571 | -0.03 (-0.24, 0.19) | 0.816 | -0.03 (-0.30, 0.24) | 0.809 |
|  | *P* for trend | 0.828 |  | 0.602 |  | 0.601 |  | 0.664 |  | 0.982 |  | 0.835 |  |
| **5-OH-IMI** | Continuous | -0.03 (-0.08, 0.02) | 0.271 | -0.15 (-0.29, -0.01) | 0.035 | -0.17 (-0.32, -0.02) | 0.022 | -0.01 (-0.09, 0.07) | 0.744 | 0.01 (-0.05, 0.06) | 0.873 | -0.05 (-0.12, 0.02) | 0.140 |
|  | Q1 | reference |  | reference |  | reference |  | reference |  | reference |  | reference |  |
|  | Q2 | 0.01 (-0.20, 0.20) | 0.993 | 0.16 (-0.38, 0.69) | 0.559 | 0.15 (-0.42, 0.73) | 0.598 | -0.05 (-0.36, 0.25) | 0.734 | -0.01 (-0.22, 0.21) | 0.947 | -0.08 (-0.35, 0.19) | 0.563 |
|  | Q3 | -0.10 (-0.30, 0.10) | 0.322 | 0.06 (-0.47, 0.60) | 0.821 | -0.02 (-0.59, 0.55) | 0.941 | 0.10 (-0.21, 0.40) | 0.530 | 0.12 (-0.09, 0.33) | 0.272 | -0.06 (-0.33, 0.21) | 0.666 |
|  | Q4 | -0.05 (-0.25, 0.14) | 0.580 | -0.19 (-0.72, 0.33) | 0.472 | -0.23 (-0.79, 0.33) | 0.425 | -0.07 (-0.37, 0.23) | 0.650 | 0.02 (-0.19, 0.23) | 0.831 | -0.08 (-0.34, 0.19) | 0.572 |
|  | *P* for trend | 0.429 |  | 0.459 |  | 0.377 |  | 0.863 |  | 0.605 |  | 0.606 |  |
| **ACE** | Continuous | 0.01 (-0.10, 0.11) | 0.933 | -0.06 (-0.35, 0.24) | 0.691 | -0.05 (-0.37, 0.26) | 0.732 | -0.09 (-0.26, 0.07) | 0.268 | -0.07 (-0.19, 0.05) | 0.238 | 0.02 (-0.12, 0.17) | 0.750 |
|  | Q1 | reference |  | reference |  | reference |  | reference |  | reference |  | reference |  |
|  | Q2 | 0.08 (-0.11, 0.27) | 0.405 | -0.34 (-0.86, 0.17) | 0.192 | -0.26 (-0.82, 0.29) | 0.349 | 0.18 (-0.12, 0.47) | 0.233 | 0.11 (-0.09, 0.32) | 0.271 | -0.01 (-0.26, 0.26) | 0.984 |
|  | Q3 | -0.02 (-0.21, 0.18) | 0.858 | 0.03 (-0.50, 0.55) | 0.924 | 0.01 (-0.56, 0.57) | 0.987 | 0.12 (-0.18, 0.42) | 0.417 | 0.11 (-0.10, 0.32) | 0.290 | 0.09 (-0.18, 0.35) | 0.525 |
|  | Q4 | 0.08 (-0.12, 0.27) | 0.444 | -0.07 (-0.59, 0.46) | 0.796 | 0.01 (-0.56, 0.57) | 0.990 | -0.11 (-0.41, 0.19) | 0.481 | -0.09 (-0.30, 0.12) | 0.392 | 0.01 (-0.25, 0.27) | 0.941 |
|  | *P* for trend | 0.677 |  | 0.860 |  | 0.767 |  | 0.454 |  | 0.434 |  | 0.778 |  |
| **CLO** | Continuous | 0.03 (-0.02, 0.08) | 0.286 | -0.09 (-0.23, 0.06) | 0.244 | -0.05 (-0.21, 0.1) | 0.519 | 0.01 (-0.07, 0.1) | 0.752 | 0.01 (-0.05, 0.06) | 0.847 | -0.02 (-0.1, 0.05) | 0.530 |
|  | Q1 | reference |  | reference |  | reference |  | reference |  | reference |  | reference |  |
|  | Q2 | 0.11 (-0.08, 0.30) | 0.262 | 0.15 (-0.37, 0.68) | 0.560 | 0.27 (-0.30, 0.83) | 0.351 | 0.04 (-0.26, 0.34) | 0.796 | 0.01 (-0.21, 0.22) | 0.973 | 0.06 (-0.2-, 0.32) | 0.654 |
|  | Q3 | 0.11 (-0.09, 0.31) | 0.272 | -0.05 (-0.58, 0.48) | 0.849 | 0.08 (-0.49, 0.66) | 0.774 | -0.06 (-0.37, 0.25) | 0.701 | -0.06 (-0.27, 0.16) | 0.610 | -0.15 (-0.41, 0.12) | 0.281 |
|  | Q4 | 0.19 (-0.01, 0.39) | 0.054 | -0.41 (-0.94, 0.12) | 0.125 | -0.21 (-0.78, 0.36) | 0.472 | -0.03 (-0.34, 0.28) | 0.845 | -0.06 (-0.27, 0.16) | 0.597 | -0.03 (-0.30, 0.23) | 0.805 |
|  | *P* for trend | 0.066 |  | 0.088 |  | 0.374 |  | 0.694 |  | 0.497 |  | 0.471 |  |
| **IMI** | Continuous | 0.04 (-0.02, 0.11) | 0.206 | -0.05 (-0.24, 0.13) | 0.555 | -0.01 (-0.21, 0.18) | 0.892 | 0.02 (-0.09, 0.12) | 0.723 | -0.0 (-0.08, 0.07) | 0.941 | -0.03 (-0.12, 0.06) | 0.503 |
|  | Q1 | reference |  | reference |  | reference |  | reference |  | reference |  | reference |  |
|  | Q2 | 0.14 (-0.05, 0.33) | 0.155 | -0.11 (-0.64, 0.42) | 0.682 | 0.04 (-0.52, 0.61) | 0.881 | 0.04 (-0.26, 0.34) | 0.783 | 0.01 (-0.20, 0.22) | 0.930 | -0.07 (-0.33, 0.20) | 0.616 |
|  | Q3 | 0.16 (-0.03, 0.36) | 0.103 | -0.16 (-0.70, 0.38) | 0.559 | 0.02 (-0.56, 0.59) | 0.953 | 0.11 (-0.20, 0.42) | 0.474 | 0.09 (-0.12, 0.31) | 0.390 | 0.05 (-0.22, 0.31) | 0.739 |
|  | Q4 | 0.05 (-0.14, 0.25) | 0.587 | -0.24 (-0.77, 0.29) | 0.373 | -0.19 (-0.76, 0.38) | 0.513 | 0.01 (-0.30, 0.31) | 0.956 | -0.04 (-0.25, 0.18) | 0.731 | -0.06 (-0.33, 0.20) | 0.641 |
|  | *P* for trend | 0.576 |  | 0.366 |  | 0.510 |  | 0.864 |  | 0.905 |  | 0.831 |  |
| **IMIT** | Continuous | 0.01 (-0.07, 0.09) | 0.882 | 0.13 (-0.09, 0.34) | 0.251 | 0.12 (-0.11, 0.36) | 0.301 | -0.01 (-0.13, 0.12) | 0.934 | 0.01 (-0.08, 0.10) | 0.834 | 0.06 (-0.05, 0.17) | 0.285 |
|  | Q1 | reference |  | reference |  | reference |  | reference |  | reference |  | reference |  |
|  | Q2 | -0.05 (-0.25, 0.15) | 0.627 | -0.03 (-0.57, 0.50) | 0.905 | -0.08 (-0.65, 0.49) | 0.783 | 0.08 (-0.22, 0.39) | 0.588 | 0.04 (-0.17, 0.26) | 0.698 | -0.11 (-0.38, 0.16) | 0.421 |
|  | Q3 | -0.05 (-0.25, 0.14) | 0.595 | -0.07 (-0.60, 0.47) | 0.804 | -0.11 (-0.69, 0.46) | 0.702 | 0.12 (-0.18, 0.43) | 0.431 | 0.04 (-0.18, 0.26) | 0.712 | -0.18 (-0.44, 0.09) | 0.189 |
|  | Q4 | 0.01 (-0.18, 0.21) | 0.882 | 0.20 (-0.33, 0.74) | 0.454 | 0.22 (-0.36, 0.79) | 0.460 | -0.07 (-0.38, 0.23) | 0.631 | -0.03 (-0.25, 0.18) | 0.774 | 0.01 (-0.26, 0.27) | 0.963 |
|  | *P* for trend | 0.901 |  | 0.500 |  | 0.503 |  | 0.706 |  | 0.782 |  | 0.907 |  |
| **Semen** |  |  |  |  |  |  |  |  |  |  |  |  |  |
| **DN** | Continuous | 0.21 (0.03, 0.39) | 0.020 | 0.22 (-0.28, 0.71) | 0.388 | 0.44 (-0.09, 0.96) | 0.102 | -0.09 (-0.37, 0.19) | 0.532 | -0.05 (-0.25, 0.15) | 0.629 | 0.07 (-0.17, 0.32) | 0.551 |
|  | Q1 | reference |  | reference |  | reference |  | reference |  | reference |  | reference |  |
|  | Q2 | -0.01 (-0.21, 0.18) | 0.91 | 0.43 (-0.11, 0.96) | 0.117 | 0.42 (-0.16, 0.99) | 0.153 | 0.08 (-0.23, 0.38) | 0.616 | 0.02 (-0.19, 0.24) | 0.840 | -0.05 (-0.31, 0.22) | 0.737 |
|  | Q3 | 0.07 (-0.13, 0.27) | 0.485 | 0.16 (-0.38, 0.71) | 0.551 | 0.25 (-0.33, 0.84) | 0.396 | -0.14 (-0.45, 0.17) | 0.384 | -0.06 (-0.28, 0.16) | 0.604 | 0.01 (-0.27, 0.27) | 0.999 |
|  | Q4 | 0.15 (-0.05, 0.35) | 0.148 | 0.15 (-0.39, 0.7) | 0.579 | 0.31 (-0.27, 0.9) | 0.292 | -0.12 (-0.43, 0.2) | 0.459 | -0.08 (-0.3, 0.14) | 0.465 | 0.06 (-0.21, 0.33) | 0.664 |
|  | *P* for trend | 0.106 |  | 0.810 |  | 0.405 |  | 0.270 |  | 0.364 |  | 0.607 |  |
| **DN-IMI** | Continuous | 0.04 (-0.02, 0.1) | 0.187 | -0.15 (-0.31, 0.01) | 0.068 | -0.11 (-0.28, 0.06) | 0.210 | -0.02 (-0.12, 0.07) | 0.621 | -0.01 (-0.07, 0.06) | 0.826 | -0.04 (-0.12, 0.05) | 0.389 |
|  | Q1 | reference |  | reference |  | reference |  | reference |  | reference |  | reference |  |
|  | Q2 | 0.12 (-0.07, 0.31) | 0.220 | -0.04 (-0.56, 0.48) | 0.879 | 0.06 (-0.49, 0.62) | 0.825 | -0.01 (-0.31, 0.3) | 0.959 | 0.02 (-0.19, 0.23) | 0.857 | -0.09 (-0.35, 0.18) | 0.516 |
|  | Q3 | 0.13 (-0.06, 0.33) | 0.170 | 0.09 (-0.43, 0.61) | 0.725 | 0.21 (-0.34, 0.76) | 0.453 | 0.11 (-0.19, 0.41) | 0.484 | 0.07 (-0.14, 0.28) | 0.511 | 0.01 (-0.26, 0.27) | 0.963 |
|  | Q4 | 0.06 (-0.14, 0.25) | 0.554 | -0.51 (-1.03, 0.01) | 0.054 | -0.46 (-1.01, 0.10) | 0.105 | -0.06 (-0.36, 0.24) | 0.700 | -0.01 (-0.22, 0.20) | 0.904 | -0.13 (-0.40, 0.13) | 0.314 |
|  | *P* for trend | 0.527 |  | 0.097 |  | 0.177 |  | 0.882 |  | 0.977 |  | 0.443 |  |
| **IMI-urea** | Continuous | -0.03 (-0.11, 0.05) | 0.438 | -0.22 (-0.43, -0.01) | 0.038 | -0.26 (-0.48, -0.03) | 0.027 | -0.08 (-0.2, 0.04) | 0.212 | -0.09 (-0.18, -0.01) | 0.034 | -0.09 (-0.20, 0.01) | 0.084 |
|  | Q1 | reference |  | reference |  | reference |  | reference |  | reference |  | reference |  |
|  | Q2 | -0.05 (-0.25, 0.14) | 0.587 | -0.45 (-0.98, 0.08) | 0.097 | -0.51 (-1.08, 0.06) | 0.077 | 0.11 (-0.20, 0.41) | 0.483 | -0.03 (-0.24, 0.18) | 0.770 | -0.10 (-0.36, 0.17) | 0.482 |
|  | Q3 | -0.08 (-0.27, 0.12) | 0.445 | -0.32 (-0.85, 0.22) | 0.243 | -0.40 (-0.96, 0.17) | 0.172 | 0.11 (-0.20, 0.41) | 0.48 | -0.02 (-0.23, 0.20) | 0.879 | -0.08 (-0.35, 0.19) | 0.556 |
|  | Q4 | -0.05 (-0.26, 0.16) | 0.622 | -0.44 (-1.00, 0.12) | 0.122 | -0.49 (-1.09, 0.1) | 0.105 | -0.19 (-0.51, 0.13) | 0.236 | -0.23 (-0.46, -0.01) | 0.039 | -0.17 (-0.45, 0.11) | 0.229 |
|  | *P* for trend | 0.550 |  | 0.165 |  | 0.132 |  | 0.344 |  | 0.070 |  | 0.265 |  |
| **ACE** | Continuous | 0.06 (0.01, 0.10) | 0.010 | -0.03 (-0.15, 0.10) | 0.676 | 0.03 (-0.10, 0.16) | 0.618 | -0.01 (-0.08, 0.06) | 0.777 | -0.03 (-0.08, 0.02) | 0.255 | -0.0 (-0.06, 0.06) | 0.917 |
|  | Q1 | reference |  | reference |  | reference |  | reference |  | reference |  | reference |  |
|  | Q2 | 0.07 (-0.12, 0.25) | 0.466 | 0.06 (-0.46, 0.59) | 0.810 | 0.13 (-0.44, 0.70) | 0.654 | 0.04 (-0.26, 0.34) | 0.788 | -0.04 (-0.25, 0.17) | 0.728 | -0.06 (-0.32, 0.21) | 0.668 |
|  | Q3 | 0.34 (0.16, 0.53) | <0.001 | -0.22 (-0.75, 0.30) | 0.404 | 0.12 (-0.45, 0.69) | 0.673 | -0.03 (-0.33, 0.27) | 0.849 | -0.07 (-0.28, 0.14) | 0.514 | 0.03 (-0.23, 0.29) | 0.819 |
|  | Q4 | 0.20 (0.02, 0.38) | 0.033 | -0.17 (-0.69, 0.35) | 0.520 | 0.03 (-0.54, 0.59) | 0.924 | 0.01 (-0.30, 0.30) | 0.992 | -0.09 (-0.30, 0.13) | 0.423 | 0.10 (-0.17, 0.36) | 0.474 |
|  | *P* for trend | 0.004 |  | 0.339 |  | 0.929 |  | 0.891 |  | 0.387 |  | 0.376 |  |

**Note:** Natural log-transformed exposure variables were divided into quartiles (Q1–Q4), with the lowest quartile (Q1) serving as the reference group. Models were adjusted for potential confounders, including age, body mass index (BMI), education level, smoking status, season of sample collection, abstinence duration, and occupational exposure likelihood. Effect estimates are presented as regression coefficients with 95% confidence intervals. *P* for trend was calculated by assigning the median value of each quartile to a continuous variable and modeling it as a linear term.

Table S8. Associations of NEO concentrations in urine (n = 179) and semen (n = 173) with semen quality parameters after further including sperm volume as a covariate in the models.

| **Exposure** |  | **Sperm concentration** | | **Sperm count** | | **Progressive motility** | | **Total motility** | | **Sperm morphology** | |
| --- | --- | --- | --- | --- | --- | --- | --- | --- | --- | --- | --- |
|  |  | **β (95% CI)** | ***P*** | **β (95% CI)** | ***P*** | **β (95% CI)** | ***P*** | **β (95% CI)** | ***P*** | **β (95% CI)** | ***P*** |
| **Urine** |  |  |  |  |  |  |  |  |  |  |  |
| **DN** | Continuous | -0.08 (-0.35, 0.20) | 0.588 | -0.09 (-0.37, 0.18) | 0.503 | -0.18 (-0.34, -0.01) | 0.039 | -0.12 (-0.25, 0.01) | 0.066 | 0.01 (-0.13, 0.14) | 0.953 |
|  | Q1 | reference |  | reference |  | reference |  | reference |  | reference |  |
|  | Q2 | 0.15 (-0.32, 0.62) | 0.536 | 0.13 (-0.34, 0.61) | 0.579 | 0.04 (-0.24, 0.33) | 0.773 | -0.03 (-0.24, 0.19) | 0.798 | 0.01 (-0.23, 0.24) | 0.956 |
|  | Q3 | -0.01 (-0.49, 0.47) | 0.969 | -0.04 (-0.53, 0.44) | 0.855 | -0.15 (-0.44, 0.14) | 0.306 | -0.12 (-0.34, 0.10) | 0.27 | -0.04 (-0.29, 0.20) | 0.718 |
|  | Q4 | 0.02 (-0.45, 0.49) | 0.940 | -0.01 (-0.48, 0.46) | 0.960 | -0.28 (-0.57, -0.01) | 0.048 | -0.19 (-0.41, 0.02) | 0.078 | -0.02 (-0.25, 0.22) | 0.899 |
|  | *P* for trend | 0.911 |  | 0.798 |  | 0.025 |  | 0.055 |  | 0.807 |  |
| **UF** | Continuous | -0.13 (-0.27, 0.01) | 0.065 | -0.13 (-0.27, 0.01) | 0.077 | -0.05 (-0.14, 0.04) | 0.253 | -0.04 (-0.11, 0.03) | 0.228 | -0.04 (-0.11, 0.04) | 0.325 |
|  | Q1 | reference |  | reference |  | reference |  | reference |  | reference |  |
|  | Q2 | -0.09 (-0.56, 0.37) | 0.689 | -0.06 (-0.52, 0.41) | 0.811 | -0.06 (-0.34, 0.22) | 0.685 | -0.07 (-0.28, 0.14) | 0.502 | -0.08 (-0.31, 0.16) | 0.526 |
|  | Q3 | -0.39 (-0.87, 0.08) | 0.100 | -0.42 (-0.89, 0.06) | 0.083 | -0.24 (-0.53, 0.04) | 0.097 | -0.18 (-0.40, 0.03) | 0.098 | -0.02 (-0.26, 0.22) | 0.874 |
|  | Q4 | -0.26 (-0.73, 0.20) | 0.266 | -0.24 (-0.7, 0.23) | 0.318 | -0.24 (-0.52, 0.05) | 0.103 | -0.22 (-0.44, -0.01) | 0.039 | -0.07 (-0.31, 0.16) | 0.548 |
|  | *P* for trend | 0.149 |  | 0.160 |  | 0.053 |  | 0.023 |  | 0.662 |  |
| **DN-IMI-olefin** | Continuous | -0.12 (-0.29, 0.06) | 0.190 | -0.12 (-0.29, 0.06) | 0.187 | -0.10 (-0.20, 0.01) | 0.076 | -0.08 (-0.16, 0.01) | 0.052 | -0.07 (-0.16, 0.02) | 0.117 |
|  | Q1 | reference |  | reference |  | reference |  | reference |  | reference |  |
|  | Q2 | -0.03 (-0.50, 0.43) | 0.885 | -0.02 (-0.49, 0.44) | 0.923 | 0.02 (-0.27, 0.31) | 0.890 | 0.01 (-0.20, 0.23) | 0.894 | 0.02 (-0.21, 0.25) | 0.864 |
|  | Q3 | 0.11 (-0.36, 0.57) | 0.653 | 0.10 (-0.37, 0.57) | 0.669 | -0.06 (-0.35, 0.22) | 0.662 | -0.01 (-0.23, 0.2) | 0.908 | 0.09 (-0.14, 0.33) | 0.427 |
|  | Q4 | -0.18 (-0.66, 0.29) | 0.450 | -0.17 (-0.65, 0.30) | 0.473 | -0.13 (-0.42, 0.16) | 0.384 | -0.13 (-0.35, 0.09) | 0.244 | -0.10 (-0.34, 0.14) | 0.411 |
|  | *P* for trend | 0.601 |  | 0.610 |  | 0.314 |  | 0.238 |  | 0.568 |  |
| **DM-ACE** | Continuous | -0.08 (-0.20, 0.05) | 0.232 | -0.06 (-0.19, 0.06) | 0.336 | 0.02 (-0.05, 0.10) | 0.540 | 0.02 (-0.04, 0.08) | 0.555 | -0.01 (-0.08, 0.05) | 0.686 |
|  | Q1 | reference |  | reference |  | reference |  | reference |  | reference |  |
|  | Q2 | -0.05 (-0.51, 0.41) | 0.819 | -0.01 (-0.47, 0.45) | 0.977 | 0.14 (-0.14, 0.42) | 0.328 | 0.12 (-0.09, 0.34) | 0.247 | 0.26 (0.03, 0.49) | 0.025 |
|  | Q3 | 0.02 (-0.44, 0.48) | 0.944 | 0.06 (-0.40, 0.53) | 0.782 | 0.17 (-0.11, 0.45) | 0.240 | 0.12 (-0.10, 0.33) | 0.284 | 0.02 (-0.21, 0.24) | 0.876 |
|  | Q4 | -0.33 (-0.80, 0.13) | 0.160 | -0.26 (-0.73, 0.20) | 0.266 | -0.03 (-0.32, 0.25) | 0.826 | -0.03 (-0.25, 0.18) | 0.765 | -0.06 (-0.29, 0.17) | 0.619 |
|  | *P* for trend | 0.223 |  | 0.349 |  | 0.916 |  | 0.789 |  | 0.288 |  |
| **DN-IMI** | Continuous | -0.03 (-0.1, 0.05) | 0.494 | -0.02 (-0.1, 0.05) | 0.549 | 0.01 (-0.04, 0.05) | 0.906 | 0.02 (-0.02, 0.05) | 0.348 | 0.01 (-0.03, 0.05) | 0.550 |
|  | Q1 | reference |  | reference |  | reference |  | reference |  | reference |  |
|  | Q2 | -0.07 (-0.54, 0.4) | 0.759 | -0.08 (-0.55, 0.39) | 0.727 | 0.06 (-0.23, 0.34) | 0.699 | 0.07 (-0.15, 0.29) | 0.511 | -0.14 (-0.37, 0.10) | 0.256 |
|  | Q3 | 0.04 (-0.43, 0.51) | 0.861 | 0.08 (-0.39, 0.54) | 0.751 | 0.05 (-0.24, 0.33) | 0.754 | 0.09 (-0.13, 0.30) | 0.428 | 0.01 (-0.22, 0.25) | 0.909 |
|  | Q4 | -0.23 (-0.70, 0.24) | 0.329 | -0.23 (-0.70, 0.24) | 0.336 | -0.01 (-0.29, 0.29) | 0.998 | 0.10 (-0.12, 0.31) | 0.386 | -0.01 (-0.24, 0.23) | 0.939 |
|  | *P* for trend | 0.450 |  | 0.498 |  | 0.989 |  | 0.378 |  | 0.745 |  |
| **IMI-urea** | Continuous | -0.10 (-0.27, 0.06) | 0.222 | -0.11 (-0.27, 0.06) | 0.208 | -0.08 (-0.18, 0.02) | 0.124 | -0.04 (-0.12, 0.04) | 0.289 | -0.03 (-0.12, 0.05) | 0.411 |
|  | Q1 | reference |  | reference |  | reference |  | reference |  | reference |  |
|  | Q2 | -0.69 (-1.15, -0.22) | 0.004 | -0.68 (-1.14, -0.21) | 0.005 | 0.04 (-0.24, 0.33) | 0.762 | 0.04 (-0.17, 0.26) | 0.687 | -0.12 (-0.36, 0.12) | 0.327 |
|  | Q3 | -0.35 (-0.81, 0.11) | 0.137 | -0.34 (-0.81, 0.12) | 0.146 | 0.07 (-0.21, 0.36) | 0.623 | 0.09 (-0.12, 0.31) | 0.408 | -0.07 (-0.31, 0.17) | 0.559 |
|  | Q4 | -0.34 (-0.80, 0.12) | 0.143 | -0.33 (-0.79, 0.13) | 0.154 | -0.26 (-0.54, 0.03) | 0.076 | -0.17 (-0.38, 0.05) | 0.124 | -0.16 (-0.39, 0.08) | 0.183 |
|  | *P* for trend | 0.315 |  | 0.329 |  | 0.107 |  | 0.188 |  | 0.245 |  |
| **5-OH-IMI** | Continuous | -0.13 (-0.25, -0.01) | 0.037 | -0.13 (-0.25, -0.01) | 0.042 | -0.01 (-0.09, 0.06) | 0.741 | 0.0 (-0.06, 0.06) | 0.955 | -0.07 (-0.13, -0.01) | 0.024 |
|  | Q1 | reference |  | reference |  | reference |  | reference |  | reference |  |
|  | Q2 | 0.07 (-0.39, 0.54) | 0.759 | 0.08 (-0.39, 0.54) | 0.746 | -0.01 (-0.29, 0.28) | 0.992 | -0.02 (-0.24, 0.19) | 0.820 | -0.12 (-0.35, 0.11) | 0.299 |
|  | Q3 | 0.10 (-0.37, 0.57) | 0.665 | 0.14 (-0.33, 0.61) | 0.555 | 0.15 (-0.14, 0.44) | 0.300 | 0.14 (-0.07, 0.36) | 0.197 | 0.01 (-0.23, 0.24) | 0.969 |
|  | Q4 | -0.22 (-0.68, 0.25) | 0.355 | -0.19 (-0.66, 0.27) | 0.416 | -0.04 (-0.32, 0.25) | 0.807 | 0.01 (-0.20, 0.23) | 0.926 | -0.11 (-0.35, 0.12) | 0.336 |
|  | *P* for trend | 0.407 |  | 0.495 |  | 0.941 |  | 0.600 |  | 0.538 |  |
| **ACE** | Continuous | -0.08 (-0.34, 0.19) | 0.563 | -0.09 (-0.35, 0.17) | 0.509 | -0.08 (-0.24, 0.08) | 0.352 | -0.06 (-0.18, 0.06) | 0.346 | 0.03 (-0.10, 0.17) | 0.616 |
|  | Q1 | reference |  | reference |  | reference |  | reference |  | reference |  |
|  | Q2 | -0.34 (-0.8, 0.11) | 0.140 | -0.32 (-0.78, 0.14) | 0.168 | 0.03 (-0.25, 0.31) | 0.827 | -0.01 (-0.22, 0.20) | 0.931 | -0.12 (-0.35, 0.11) | 0.313 |
|  | Q3 | -0.11 (-0.57, 0.35) | 0.643 | -0.13 (-0.59, 0.34) | 0.592 | 0.07 (-0.22, 0.35) | 0.631 | 0.03 (-0.18, 0.25) | 0.752 | 0.10 (-0.13, 0.33) | 0.392 |
|  | Q4 | -0.22 (-0.68, 0.25) | 0.361 | -0.22 (-0.69, 0.24) | 0.342 | -0.07 (-0.36, 0.21) | 0.609 | -0.07 (-0.29, 0.15) | 0.527 | 0.03 (-0.20, 0.26) | 0.817 |
|  | *P* for trend | 0.586 |  | 0.526 |  | 0.689 |  | 0.636 |  | 0.417 |  |
| **CLO** | Continuous | -0.09 (-0.22, 0.04) | 0.157 | -0.10 (-0.22, 0.03) | 0.148 | 0.02 (-0.06, 0.10) | 0.606 | 0.01 (-0.05, 0.07) | 0.712 | -0.01 (-0.07, 0.06) | 0.779 |
|  | Q1 | reference |  | reference |  | reference |  | reference |  | reference |  |
|  | Q2 | -0.08 (-0.55, 0.38) | 0.721 | -0.06 (-0.52, 0.41) | 0.808 | 0.0 (-0.29, 0.29) | 0.998 | -0.02 (-0.24, 0.20) | 0.858 | 0.07 (-0.16, 0.31) | 0.538 |
|  | Q3 | -0.20 (-0.68, 0.28) | 0.420 | -0.16 (-0.64, 0.32) | 0.516 | 0.01 (-0.28, 0.31) | 0.927 | 0.02 (-0.21, 0.24) | 0.875 | -0.12 (-0.36, 0.12) | 0.322 |
|  | Q4 | -0.47 (-0.95, 0.01) | 0.052 | -0.46 (-0.94, 0.02) | 0.059 | 0.07 (-0.22, 0.37) | 0.632 | 0.04 (-0.18, 0.26) | 0.736 | -0.02 (-0.26, 0.22) | 0.856 |
|  | *P* for trend | 0.045 |  | 0.052 |  | 0.623 |  | 0.668 |  | 0.513 |  |
| **IMI** | Continuous | -0.03 (-0.19, 0.13) | 0.712 | -0.02 (-0.17, 0.14) | 0.845 | -0.01 (-0.11, 0.08) | 0.795 | -0.02 (-0.10, 0.05) | 0.523 | -0.05 (-0.13, 0.03) | 0.191 |
|  | Q1 | reference |  | reference |  | reference |  | reference |  | reference |  |
|  | Q2 | 0.04 (-0.42, 0.51) | 0.850 | 0.08 (-0.39, 0.54) | 0.739 | -0.13 (-0.41, 0.16) | 0.370 | -0.11 (-0.32, 0.11) | 0.326 | -0.12 (-0.35, 0.11) | 0.310 |
|  | Q3 | -0.15 (-0.62, 0.32) | 0.535 | -0.11 (-0.58, 0.36) | 0.652 | -0.14 (-0.43, 0.15) | 0.345 | -0.09 (-0.31, 0.13) | 0.409 | 0.02 (-0.21, 0.25) | 0.858 |
|  | Q4 | -0.18 (-0.64, 0.28) | 0.446 | -0.15 (-0.61, 0.32) | 0.533 | -0.11 (-0.39, 0.18) | 0.449 | -0.13 (-0.34, 0.08) | 0.233 | -0.17 (-0.4, 0.06) | 0.146 |
|  | *P* for trend | 0.327 |  | 0.401 |  | 0.467 |  | 0.278 |  | 0.306 |  |
| **IMIT** | Continuous | 0.12 (-0.07, 0.31) | 0.227 | 0.11 (-0.08, 0.31) | 0.249 | -0.01 (-0.13, 0.11) | 0.869 | 0.02 (-0.07, 0.11) | 0.675 | 0.03 (-0.07, 0.13) | 0.528 |
|  | Q1 | reference |  | reference |  | reference |  | reference |  | reference |  |
|  | Q2 | 0.29 (-0.18, 0.77) | 0.227 | 0.32 (-0.16, 0.80) | 0.186 | 0.18 (-0.11, 0.47) | 0.221 | 0.14 (-0.08, 0.37) | 0.201 | -0.07 (-0.31, 0.17) | 0.577 |
|  | Q3 | -0.13 (-0.60, 0.33) | 0.578 | -0.10 (-0.56, 0.37) | 0.686 | 0.18 (-0.10, 0.47) | 0.208 | 0.14 (-0.07, 0.36) | 0.194 | -0.16 (-0.39, 0.07) | 0.178 |
|  | Q4 | 0.25 (-0.23, 0.72) | 0.305 | 0.26 (-0.21, 0.74) | 0.279 | -0.05 (-0.34, 0.24) | 0.731 | 0.02 (-0.20, 0.24) | 0.872 | -0.02 (-0.26, 0.21) | 0.840 |
|  | *P* for trend | 0.695 |  | 0.646 |  | 0.758 |  | 0.871 |  | 0.650 |  |
| **Semen** |  |  |  |  |  |  |  |  |  |  |  |
| **DN** | Continuous | 0.05 (-0.38, 0.48) | 0.817 | 0.06 (-0.38, 0.50) | 0.785 | -0.07 (-0.33, 0.19) | 0.611 | -0.04 (-0.22, 0.15) | 0.688 | 0.03 (-0.18, 0.25) | 0.763 |
|  | Q1 | reference |  | reference |  | reference |  | reference |  | reference |  |
|  | Q2 | 0.36 (-0.11, 0.83) | 0.129 | 0.34 (-0.13, 0.81) | 0.150 | 0.18 (-0.10, 0.46) | 0.207 | 0.09 (-0.11, 0.29) | 0.371 | 0.01 (-0.23, 0.24) | 0.944 |
|  | Q3 | 0.15 (-0.33, 0.63) | 0.535 | 0.13 (-0.35, 0.61) | 0.595 | -0.01 (-0.29, 0.29) | 0.992 | 0.03 (-0.18, 0.23) | 0.789 | 0.06 (-0.18, 0.30) | 0.624 |
|  | Q4 | 0.07 (-0.43, 0.56) | 0.787 | 0.03 (-0.46, 0.53) | 0.894 | -0.06 (-0.35, 0.24) | 0.694 | -0.04 (-0.25, 0.18) | 0.732 | 0.03 (-0.22, 0.28) | 0.837 |
|  | *P* for trend | 0.988 |  | 0.906 |  | 0.471 |  | 0.624 |  | 0.749 |  |
| **DN-IMI** | Continuous | -0.17 (-0.31, -0.03) | 0.018 | -0.17 (-0.31, -0.03) | 0.021 | -0.01 (-0.09, 0.08) | 0.917 | -0.01 (-0.06, 0.06) | 0.966 | -0.03 (-0.10, 0.04) | 0.376 |
|  | Q1 | reference |  | reference |  | reference |  | reference |  | reference |  |
|  | Q2 | -0.03 (-0.49, 0.43) | 0.903 | -0.02 (-0.48, 0.44) | 0.936 | -0.03 (-0.31, 0.25) | 0.842 | -0.01 (-0.2, 0.20) | 0.978 | -0.07 (-0.30, 0.17) | 0.571 |
|  | Q3 | -0.07 (-0.52, 0.39) | 0.772 | -0.07 (-0.53, 0.39) | 0.772 | 0.19 (-0.09, 0.47) | 0.175 | 0.14 (-0.06, 0.33) | 0.173 | -0.01 (-0.23, 0.23) | 0.996 |
|  | Q4 | -0.56 (-1.02, -0.10) | 0.017 | -0.56 (-1.02, -0.10) | 0.018 | -0.07 (-0.35, 0.21) | 0.617 | -0.05 (-0.25, 0.15) | 0.621 | -0.11 (-0.35, 0.12) | 0.336 |
|  | *P* for trend | 0.021 |  | 0.022 |  | 0.989 |  | 0.957 |  | 0.452 |  |
| **IMI-urea** | Continuous | -0.21 (-0.4, -0.02) | 0.030 | -0.22 (-0.41, -0.03) | 0.023 | -0.07 (-0.19, 0.04) | 0.202 | -0.09 (-0.17, -0.01) | 0.037 | -0.09 (-0.18, 0.01) | 0.079 |
|  | Q1 | reference |  | reference |  | reference |  | reference |  | reference |  |
|  | Q2 | -0.58 (-1.04, -0.11) | 0.016 | -0.61 (-1.07, -0.14) | 0.012 | -0.06 (-0.35, 0.22) | 0.654 | -0.16 (-0.36, 0.04) | 0.122 | -0.15 (-0.39, 0.08) | 0.202 |
|  | Q3 | -0.37 (-0.83, 0.10) | 0.123 | -0.36 (-0.83, 0.10) | 0.127 | 0.03 (-0.25, 0.31) | 0.827 | -0.07 (-0.27, 0.13) | 0.493 | -0.05 (-0.29, 0.18) | 0.657 |
|  | Q4 | -0.48 (-0.98, 0.03) | 0.063 | -0.51 (-1.02, -0.01) | 0.047 | -0.24 (-0.54, 0.07) | 0.124 | -0.26 (-0.47, -0.04) | 0.021 | -0.19 (-0.45, 0.06) | 0.142 |
|  | *P* for trend | 0.104 |  | 0.090 |  | 0.266 |  | 0.060 |  | 0.261 |  |
| **ACE** | Continuous | -0.05 (-0.16, 0.06) | 0.377 | -0.06 (-0.17, 0.06) | 0.332 | -0.01 (-0.07, 0.06) | 0.855 | -0.02 (-0.07, 0.02) | 0.324 | 0.01 (-0.05, 0.06) | 0.902 |
|  | Q1 | reference |  | reference |  | reference |  | reference |  | reference |  |
|  | Q2 | 0.10 (-0.37, 0.56) | 0.681 | 0.07 (-0.40, 0.53) | 0.780 | 0.08 (-0.20, 0.36) | 0.592 | -0.01 (-0.21, 0.19) | 0.916 | -0.03 (-0.27, 0.20) | 0.769 |
|  | Q3 | -0.32 (-0.8, 0.16) | 0.184 | -0.31 (-0.80, 0.17) | 0.201 | -0.02 (-0.31, 0.27) | 0.887 | -0.06 (-0.27, 0.14) | 0.540 | 0.11 (-0.14, 0.35) | 0.390 |
|  | Q4 | -0.25 (-0.73, 0.22) | 0.300 | -0.28 (-0.76, 0.19) | 0.243 | 0.03 (-0.25, 0.32) | 0.812 | -0.05 (-0.26, 0.15) | 0.611 | 0.11 (-0.13, 0.34) | 0.387 |
|  | *P* for trend | 0.146 |  | 0.125 |  | 0.964 |  | 0.532 |  | 0.255 |  |

**Note:** Natural log-transformed exposure variables were divided into quartiles (Q1–Q4), with the lowest quartile (Q1) serving as the reference group. Models were adjusted for potential confounders, including age, body mass index (BMI), education level, smoking status, season of sample collection, abstinence duration, occupational exposure likelihood and sperm volume. Effect estimates are presented as regression coefficients with 95% confidence intervals. *P* for trend was calculated by assigning the median value of each quartile to a continuous variable and modeling it as a linear term.

Table S9. Associations of urine (n = 179) and semen (n = 173) NEO concentrations with semen quality parameters after NEO concentrations were further normalized using the z-score method after natural log-transformation.

| **Seman quality** | **Exposure** | **Urine** | | **Semen** | |
| --- | --- | --- | --- | --- | --- |
|  |  | **β (95% CI)** | ***P*** | **β (95% CI)** | ***P*** |
| **Sperm volume** | **DN** | 0.04 (-0.03, 0.10) | 0.246 | 0.09 (0.03, 0.16) | 0.005 |
|  | **UF** | 0.03 (-0.03, 0.10) | 0.314 |  |  |
|  | **DN-IMI-olefin** | 0.07 (0.01, 0.13) | 0.029 |  |  |
|  | **DM-ACE** | 0.03 (-0.04, 0.09) | 0.405 |  |  |
|  | **DN-IMI** | -0.03 (-0.09, 0.04) | 0.427 | 0.04 (-0.02, 0.11) | 0.175 |
|  | **IMI-urea** | 0.02 (-0.05, 0.08) | 0.587 | -0.01 (-0.08, 0.06) | 0.746 |
|  | **5-OH-IMI** | -0.01 (-0.08, 0.05) | 0.694 |  |  |
|  | **ACE** | 0.01 (-0.05, 0.08) | 0.692 | 0.09 (0.03, 0.15) | 0.004 |
|  | **CLO** | 0.03 (-0.03, 0.10) | 0.321 |  |  |
|  | **IMI** | 0.05 (-0.01, 0.11) | 0.099 |  |  |
|  | **IMIT** | 0.02 (-0.04, 0.08) | 0.521 |  |  |
| **Sperm concentration** | **DN** | -0.04 (-0.21, 0.12) | 0.595 | 0.02 (-0.16, 0.20) | 0.793 |
|  | **UF** | -0.15 (-0.32, 0.01) | 0.066 |  |  |
|  | **DN-IMI-olefin** | -0.11 (-0.27, 0.06) | 0.200 |  |  |
|  | **DM-ACE** | -0.10 (-0.26, 0.06) | 0.231 |  |  |
|  | **DN-IMI** | -0.06 (-0.22, 0.11) | 0.490 | -0.20 (-0.36, -0.03) | 0.018 |
|  | **IMI-urea** | -0.10 (-0.27, 0.06) | 0.222 | -0.21 (-0.39, -0.02) | 0.029 |
|  | **5-OH-IMI** | -0.17 (-0.34, -0.01) | 0.036 |  |  |
|  | **ACE** | -0.05 (-0.21, 0.12) | 0.565 | -0.07 (-0.23, 0.10) | 0.410 |
|  | **CLO** | -0.12 (-0.29, 0.05) | 0.161 |  |  |
|  | **IMI** | -0.03 (-0.19, 0.13) | 0.716 |  |  |
|  | **IMIT** | 0.10 (-0.06, 0.26) | 0.225 |  |  |
| **Sperm count** | **DN** | -0.01 (-0.19, 0.17) | 0.913 | 0.12 (-0.07, 0.31) | 0.227 |
|  | **UF** | -0.12 (-0.30, 0.06) | 0.180 |  |  |
|  | **DN-IMI-olefin** | -0.04 (-0.22, 0.14) | 0.648 |  |  |
|  | **DM-ACE** | -0.07 (-0.25, 0.10) | 0.422 |  |  |
|  | **DN-IMI** | -0.08 (-0.25, 0.10) | 0.388 | -0.16 (-0.34, 0.02) | 0.075 |
|  | **IMI-urea** | -0.10 (-0.27, 0.08) | 0.286 | -0.22 (-0.42, -0.02) | 0.033 |
|  | **5-OH-IMI** | -0.18 (-0.35, -0.01) | 0.044 |  |  |
|  | **ACE** | -0.04 (-0.21, 0.14) | 0.664 | 0.01 (-0.17, 0.19) | 0.901 |
|  | **CLO** | -0.08 (-0.25, 0.10) | 0.404 |  |  |
|  | **IMI** | 0.01 (-0.16, 0.19) | 0.894 |  |  |
|  | **IMIT** | 0.10 (-0.07, 0.28) | 0.234 |  |  |
| **Progressive motility** | **DN** | -0.11 (-0.21, -0.01) | 0.029 | -0.04 (-0.15, 0.07) | 0.433 |
|  | **UF** | -0.06 (-0.16, 0.04) | 0.221 |  |  |
|  | **DN-IMI-olefin** | -0.10 (-0.20, 0.01) | 0.051 |  |  |
|  | **DM-ACE** | 0.03 (-0.07, 0.13) | 0.559 |  |  |
|  | **DN-IMI** | 0.01 (-0.09, 0.11) | 0.844 | -0.01 (-0.11, 0.09) | 0.828 |
|  | **IMI-urea** | -0.08 (-0.18, 0.02) | 0.117 | -0.07 (-0.19, 0.04) | 0.203 |
|  | **5-OH-IMI** | -0.02 (-0.12, 0.09) | 0.761 |  |  |
|  | **ACE** | -0.05 (-0.15, 0.05) | 0.328 | -0.02 (-0.12, 0.07) | 0.628 |
|  | **CLO** | 0.02 (-0.08, 0.12) | 0.708 |  |  |
|  | **IMI** | -0.02 (-0.12, 0.08) | 0.732 |  |  |
|  | **IMIT** | -0.01 (-0.11, 0.09) | 0.848 |  |  |
| **Total motility** | **DN** | -0.08 (-0.15, -0.01) | 0.044 | -0.03 (-0.11, 0.05) | 0.464 |
|  | **UF** | -0.05 (-0.13, 0.03) | 0.187 |  |  |
|  | **DN-IMI-olefin** | -0.09 (-0.16, -0.01) | 0.028 |  |  |
|  | **DM-ACE** | 0.02 (-0.06, 0.10) | 0.585 |  |  |
|  | **DN-IMI** | 0.04 (-0.04, 0.12) | 0.296 | -0.01 (-0.08, 0.07) | 0.859 |
|  | **IMI-urea** | -0.04 (-0.12, 0.03) | 0.272 | -0.09 (-0.17, -0.01) | 0.037 |
|  | **5-OH-IMI** | 0.01 (-0.07, 0.08) | 0.926 |  |  |
|  | **ACE** | -0.04 (-0.12, 0.04) | 0.313 | -0.05 (-0.12, 0.02) | 0.187 |
|  | **CLO** | 0.01 (-0.07, 0.08) | 0.868 |  |  |
|  | **IMI** | -0.03 (-0.11, 0.05) | 0.450 |  |  |
|  | **IMIT** | 0.01 (-0.06, 0.09) | 0.706 |  |  |
| **Sperm morphology** | **DN** | -0.01 (-0.08, 0.08) | 0.995 | 0.02 (-0.07, 0.11) | 0.713 |
|  | **UF** | -0.04 (-0.13, 0.04) | 0.305 |  |  |
|  | **DN-IMI-olefin** | -0.07 (-0.15, 0.01) | 0.100 |  |  |
|  | **DM-ACE** | -0.02 (-0.1, 0.07) | 0.676 |  |  |
|  | **DN-IMI** | 0.03 (-0.06, 0.11) | 0.525 | -0.04 (-0.12, 0.05) | 0.392 |
|  | **IMI-urea** | -0.04 (-0.12, 0.05) | 0.402 | -0.08 (-0.18, 0.01) | 0.078 |
|  | **5-OH-IMI** | -0.09 (-0.18, -0.01) | 0.024 |  |  |
|  | **ACE** | 0.02 (-0.06, 0.10) | 0.632 | 0.01 (-0.07, 0.09) | 0.844 |
|  | **CLO** | -0.01 (-0.10, 0.07) | 0.731 |  |  |
|  | **IMI** | -0.06 (-0.14, 0.03) | 0.178 |  |  |
|  | **IMIT** | 0.03 (-0.06, 0.11) | 0.536 |  |  |

**Note:** Models were adjusted for potential confounders, including age, body mass index (BMI), education level, smoking status, season of sample collection, abstinence duration and occupational exposure likelihood.

Table S10. Sensitivity analysis of the associations between urinary and seminal NEO concentrations and semen quality parameters using LOD/√2 imputation for non-detectable values.

| **Seman quality** | **Exposure** | **Urine** | | **Semen** | |
| --- | --- | --- | --- | --- | --- |
|  |  | **β (95% CI)** | ***P*** | **β (95% CI)** | ***P*** |
| **Sperm volume** | **DN** | 0.062 (-0.047, 0.170) | 0.265 | **0.217 (0.060, 0.375)** | **0.007** |
|  | **UF** | 0.036 (-0.024, 0.096) | 0.237 |  |  |
|  | **DN-IMI-olefin** | **0.075 (0.005, 0.144)** | **0.035** |  |  |
|  | **DM-ACE** | 0.019 (-0.031, 0.069) | 0.447 |  |  |
|  | **DN-IMI** | -0.011 (-0.041, 0.018) | 0.459 | 0.038 (-0.018, 0.095) | 0.180 |
|  | **IMI-urea** | 0.024 (-0.046, 0.094) | 0.498 | -0.019 (-0.099, 0.061) | 0.645 |
|  | **5-OH-IMI** | -0.010 (-0.060, 0.041) | 0.704 |  |  |
|  | **ACE** | 0.022 (-0.081, 0.124) | 0.678 | **0.065 (0.025, 0.106)** | **0.002** |
|  | **CLO** | 0.026 (-0.025, 0.076) | 0.315 |  |  |
|  | **IMI** | 0.044 (-0.019, 0.107) | 0.170 |  |  |
|  | **IMIT** | 0.031 (-0.049, 0.110) | 0.446 |  |  |
| **Sperm concentration** | **DN** | -0.076 (-0.355, 0.202) | 0.589 | 0.066 (-0.362, 0.494) | 0.761 |
|  | **UF** | -0.138 (-0.289, 0.014) | 0.075 |  |  |
|  | **DN-IMI-olefin** | -0.123 (-0.301, 0.056) | 0.178 |  |  |
|  | **DM-ACE** | -0.075 (-0.203, 0.052) | 0.245 |  |  |
|  | **DN-IMI** | -0.028 (-0.104, 0.047) | 0.457 | **-0.183 (-0.331, -0.035)** | **0.015** |
|  | **IMI-urea** | -0.108 (-0.287, 0.071) | 0.234 | **-0.234 (-0.444, -0.024)** | **0.029** |
|  | **5-OH-IMI** | **-0.128 (-0.256, -0.000)** | **0.050** |  |  |
|  | **ACE** | -0.069 (-0.330, 0.192) | 0.601 | -0.054 (-0.165, 0.058) | 0.346 |
|  | **CLO** | -0.092 (-0.221, 0.036) | 0.159 |  |  |
|  | **IMI** | -0.042 (-0.204, 0.121) | 0.613 |  |  |
|  | **IMIT** | 0.122 (-0.081, 0.325) | 0.238 |  |  |
| **Sperm count** | **DN** | -0.020 (-0.316, 0.276) | 0.896 | 0.282 (-0.176, 0.740) | 0.226 |
|  | **UF** | -0.100 (-0.262, 0.062) | 0.226 |  |  |
|  | **DN-IMI-olefin** | -0.058 (-0.249, 0.133) | 0.548 |  |  |
|  | **DM-ACE** | -0.055 (-0.191, 0.081) | 0.427 |  |  |
|  | **DN-IMI** | -0.036 (-0.116, 0.044) | 0.372 | -0.149 (-0.310, 0.011) | 0.067 |
|  | **IMI-urea** | -0.093 (-0.284, 0.097) | 0.334 | **-0.253 (-0.478, -0.027)** | **0.028** |
|  | **5-OH-IMI** | -0.134 (-0.270, 0.002) | 0.053 |  |  |
|  | **ACE** | -0.055 (-0.332, 0.223) | 0.697 | 0.007 (-0.114, 0.127) | 0.914 |
|  | **CLO** | -0.062 (-0.199, 0.075) | 0.375 |  |  |
|  | **IMI** | -0.008 (-0.181, 0.164) | 0.923 |  |  |
|  | **IMIT** | 0.126 (-0.090, 0.342) | 0.250 |  |  |
| **Progressive motility** | **DN** | **-0.193 (-0.362, -0.024)** | **0.025** | -0.100 (-0.357, 0.158) | 0.446 |
|  | **UF** | -0.062 (-0.156, 0.031) | 0.191 |  |  |
|  | **DN-IMI-olefin** | -0.109 (-0.219, 0.001) | 0.051 |  |  |
|  | **DM-ACE** | 0.023 (-0.056, 0.101) | 0.571 |  |  |
|  | **DN-IMI** | 0.004 (-0.042, 0.051) | 0.856 | -0.007 (-0.097, 0.084) | 0.886 |
|  | **IMI-urea** | -0.092 (-0.202, 0.018) | 0.100 | -0.085 (-0.213, 0.043) | 0.191 |
|  | **5-OH-IMI** | -0.015 (-0.095, 0.065) | 0.710 |  |  |
|  | **ACE** | -0.080 (-0.240, 0.081) | 0.328 | -0.017 (-0.085, 0.050) | 0.615 |
|  | **CLO** | 0.015 (-0.064, 0.095) | 0.709 |  |  |
|  | **IMI** | -0.022 (-0.122, 0.078) | 0.662 |  |  |
|  | **IMIT** | -0.019 (-0.145, 0.106) | 0.763 |  |  |
| **Total motility** | **DN** | **-0.136 (-0.265, -0.008)** | **0.038** | -0.064 (-0.249, 0.120) | 0.492 |
|  | **UF** | -0.052 (-0.123, 0.020) | 0.154 |  |  |
|  | **DN-IMI-olefin** | **-0.093 (-0.176, -0.010)** | **0.028** |  |  |
|  | **DM-ACE** | 0.014 (-0.045, 0.074) | 0.636 |  |  |
|  | **DN-IMI** | 0.018 (-0.017, 0.054) | 0.302 | -0.004 (-0.069, 0.061) | 0.907 |
|  | **IMI-urea** | -0.050 (-0.134, 0.034) | 0.239 | **-0.097 (-0.188, -0.007)** | **0.036** |
|  | **5-OH-IMI** | 0.001 (-0.059, 0.062) | 0.968 |  |  |
|  | **ACE** | -0.062 (-0.184, 0.059) | 0.313 | -0.032 (-0.080, 0.016) | 0.190 |
|  | **CLO** | 0.005 (-0.056, 0.065) | 0.874 |  |  |
|  | **IMI** | -0.032 (-0.108, 0.044) | 0.402 |  |  |
|  | **IMIT** | 0.010 (-0.085, 0.106) | 0.831 |  |  |
| **Sperm morphology** | **DN** | 0.001 (-0.140, 0.141) | 0.993 | 0.032 (-0.182, 0.246) | 0.767 |
|  | **UF** | -0.053 (-0.130, 0.023) | 0.171 |  |  |
|  | **DN-IMI-olefin** | -0.084 (-0.174, 0.006) | 0.066 |  |  |
|  | **DM-ACE** | -0.018 (-0.082, 0.047) | 0.588 |  |  |
|  | **DN-IMI** | 0.014 (-0.024, 0.052) | 0.453 | -0.040 (-0.115, 0.035) | 0.289 |
|  | **IMI-urea** | -0.064 (-0.154, 0.026) | 0.161 | -0.076 (-0.182, 0.030) | 0.157 |
|  | **5-OH-IMI** | **-0.090 (-0.154, -0.027)** | **0.006** |  |  |
|  | **ACE** | 0.021 (-0.111, 0.153) | 0.752 | 0.002 (-0.054, 0.058) | 0.935 |
|  | **CLO** | -0.007 (-0.072, 0.058) | 0.841 |  |  |
|  | **IMI** | -0.065 (-0.146, 0.016) | 0.117 |  |  |
|  | **IMIT** | 0.020 (-0.083, 0.122) | 0.706 |  |  |

**Note:** Models were adjusted for potential confounders, including age, body mass index (BMI), education level, smoking status, season of sample collection, abstinence duration and occupational exposure likelihood.
